# Validation of the Kidsights Measurement Tool: A parent-reported instrument to track children’s development at the population level

**DOI:** 10.1371/journal.pone.0324082

**Published:** 2025-06-26

**Authors:** Marcus R. Waldman, Katelyn Hepworth, Jolene Johnson, Kelsey M. Tourek, Kelly J. Jones, Yaritza Estrada Garcia, Laura M. Fritz, Abbey Siebler, Abbie Raikes

**Affiliations:** 1 Department of Biostatistics and Informatics, Colorado School of Public Health, University of Colorado Anschutz Medical Campus, Aurora, Colorado, United States of America; 2 Department of Health Promotion, College of Public Health, University of Nebraska Medical Center, Omaha, Nebraska, United States of America; 3 Munroe-Meyer Institute, Education and Child Development, University of Nebraska Medical Center, Omaha, Nebraska, United States of America; Caribbean Center for Child Neurodevelopment, GRENADA

## Abstract

Disparities in child development between groups of children arise early and reflect social inequities in early environments, geography, and other factors. To track and address these disparities, valid and reliable tools are needed that can be implemented at-scale and across populations. However, no population-level measures of child development appropriate for children from birth to age five years have been developed and validated in the United States to date. The Kidsights Measurement Tool (KMT) is a parent-report, population-level tool for children birth to age five years intended to track group-level differences in the developmental status across normative aspects of children’s motor, cognitive, language, and social/emotional development. This study reports on validation of KMT as a feasible tool that can be implemented in large-scale surveys to track disparities in early childhood development. Using a sample of *N* = 5,001 initial parent reports residing in Nebraska and across the United States, we find strong evidence that the KMT can detect disparities in child development birth to age five, as indicated by expected criterion associations with parent education and mental health, as well as child’s race and ethnicity. In addition, we found that the KMT is strongly associated with gold-standard direct observation instruments (i.e., the Bayley Scale of Infant Development and the Woodcock-Johnson) that measure similar developmental constructs both concurrently and one-year 12–24 months later. Finally, the KMT exhibits strong reliability even after controlling for age, and we find no evidence that measurement noninvariance threatens valid inferences about group difference. Taken together, our findings indicate that a parent-report measure can generate valid and useful estimates for tracking disparities in early child development at the population level.

## Introduction

Early child development lays the groundwork for lifelong health and wellbeing. Supportive early environments that promote healthy child development are associated with later adult health and well-being, including lower incidence of diseases, greater earnings, and lower risk of incarceration [1–4]. A substantial body of research outlines the biological and social mechanisms by which early development influences later health and wellbeing [5–8]. As such, investments in early childhood development yield notable long-term returns [9], as supporting healthy development early in life may be more cost-effective than mitigating the consequences of early stress and deprivation in adulthood.

Because early child development has such significant implications for later development, the impacts of early stress are visible in group-level disparities in health, learning and wellbeing throughout the life course [10]. Disparities in cognitive, language and social/emotional development outcomes in young children by socio-economic status, race/ethnicity, and geography have been extensively documented over several decades in the United States [11–13], emerging in the first year of life and persisting over time [14–16]. These disparities have been shown to be attributable to the differences in environmental supports, including access to quality childcare, economic resources, and neighborhood and community supports [15,16]. In sum, children’s development is strongly influenced by the social and economic context of their families and communities and, when these contexts are not supportive, lead to persistent group-level disparities in child development outcomes.

### Population-level tracking of early child development in the United States: Too little, too late

“Population-level tracking” refers to measuring and drawing conclusions about group-level characteristics, as opposed to drawing conclusions about individual children. Validity of claims regarding group-level characteristics depends upon a representative sampling approach as well as tools that are valid in measuring group characteristics, sufficiently reliable for this intended use, and scalable so that the data can be collected quickly and cheaply to meet large sample size requirements of population-level tracking. The purpose of this paper is to provide psychometric evidence that the Kidsights Measurement Tool (KM) is valid, reliable, and scalable for future use in large sample studies that incorporate representative sampling. As we outline below, to date, there have been few attempts to create population-level estimates of child development, despite the need for insight into early disparities across large populations of young children.

In the United States, population-level tracking of children’s development in the first five years is limited in scope and relies on a small and narrow set of indicators, especially for children birth to age three. Infant mortality, for example, is an important indicator of early inequities [17] but does not provide insight into normative child development. Yet, children from lower-income families, families with less formal education, and/or families who represent diverse racial and ethnic backgrounds are up to half a year behind their more advantaged peers in cognitive, language, and social/emotional development by the time formal schooling begins [18,19]. Given the substantive size of these disparities at the start of school, more population-level data are needed on children’s developmental outcomes earlier in life, when interventions can address emerging inequities. The lack of data is especially problematic given the rapid pace of early development and the opportunity to support healthy development through cost-effective programs such as home visiting and support for quality childcare [20].

The importance of population-level tracking of child development is underscored by US state and federal agendas, which identify indicators of early child development as key indicators of population-level health and wellbeing. The United States Center for Disease Control’s (CDC) Healthy People 2030, for example, states an objective of increasing the national proportion of children who are developmentally on track and ready to start school [21]. But progress toward this objective is not currently reported, as CDC has designated the Healthy People 2030 goal regarding children’s developmental status as “presently lacking reliable baseline data” [21].

The landscape of routinely collected population-level child development data for children five years and younger in the United States is limited to the National Survey of Children’s Health (NSCH). The NSCH is administered by the Health Resources and Services Administration using a representative sampling design and includes a parent-reported National Outcomes Measure on preschool-aged children’s development to generate population-level estimates, called Healthy and Ready to Learn (HRTL) [22,23]. The percentage of children who are HRTL is estimated based on a set of 27 parent-report items to index child development across motor development, health, early learning skills, self-regulation, and social/emotional development for children between three and six years. Results from national samples estimate that only 64.3% of children were designated HRTL. Significant disparities were found by—among other factors—the parent’s mental health status, the quality of the home learning environment, and the child’s neighborhood [23]. However, HRTL does not estimate child development for children under age three years.

The lack of workable tools for children birth to age five is a primary barrier to obtaining population-level data on child development, which is the focus of the present study. Beyond HRTL, three additional parent-report, population-level tools have been developed for use globally, the Global Scales for Early Development (GSED) for children birth to age three [24]; the Caregiver-Reported Early Development Index (CREDI) for children birth to age three [25]; and the Early Child Development Index (ECDI) for children two to five years [26], but are not at present routinely incorporated into routine household survey in the US for population-level tracking.

In sum, despite policy goals of collecting population data on infant, toddler and preschooler wellbeing and development, data on these constructs beginning at birth and extending through the start of school are largely absent from statewide and national data systems in the United States [27]. Indeed, the HRTL measure administered as part of the NSCH represents the lone population-level of child development but is limited to children aged 3–5 years. Population data on children’s development for the nation’s youngest children is currently not collected in government-led data collection efforts such as the NSCH. One barrier to the collection of population level data beginning at birth is the lack of measures that can be incorporated into routine household surveys and are appropriate for children beginning at birth and extending to school-entry. Overcoming this barrier is the focus of the present study.

### Developing measurement tools for measuring child development at the population level

Population-based tools of child development should reflect the holistic nature of early development, addressing language, cognitive, motor, and social/emotional development, and therefore must balance developmental breadth with feasibility for use at scale. The considerable time and cost of direct assessments of child development, such as the Bayley Scales of Infant Development, preclude their use at a population level, thus leading to reliance on parent report tools (e.g., CREDI; GSED; and ECDI), and requiring innovation in scale development to create accurate and reliable parent-report tools. Further, even though the tools must be feasible for use at scale, it is equally important to address the validity of population-level instruments of child development. Recommendations from American Psychological Association’s [28] *Standards for Educational and Psychological Testing* (henceforth, *Standards*) define types of validity evidence: (1) test content, (2) relations with other variables (including criterion variables and scores from concurrent instrument), (3) the sensitivity of conclusions to threats from measurement non-invariance or item misfit, and (4) score precision as indicated by errors of measurement (i.e., reliability). McCoy et al. [25] reported acceptable psychometric properties and criterion validity of the CREDI across high, middle and low-income countries. Ghandour et al. [19] similarly reported acceptable psychometric properties and criterion validity for HRTL. Validation evidence for the GSED and ECDI are still in development. However, two types of validity evidence – concurrent validity with observational measures and predictive validity demonstrating associations between parent-report and observational measures over time – have not yet been frequently reported for population-based measures of child development for children birth to age five years.

The purpose of the present study was to evaluate the validity evidence of a new parent-report measure of child development for children birth to age five living in the United States called the Kidsights Measurement Tool (KMT). When incorporated into studies designed for representative sampling, the KMT is intended to provide population-based estimates of children’s development from birth through age five, thereby addressing the present gap in holistic indicators of child development with a measure that is feasible to scale. We hypothesized that the KMT would show acceptable psychometric properties. Using the *Standards* of test reliability and validity, we assessed the KMT’s validity using a large sample (N = 5,001) including predictive validity with direct assessment administered by trained observers 12–24 months later. We also jointly addressed criterion validity and documentation of early disparities, with known correlates of children’s development, including parent education, parent anxiety and depression, the family’s socio-economic status, and exposure to experiences that may be traumatic and/or adverse to healthy development.

## Materials and methods

### Participants

Participants were adults who were responsible for the child’s care and reported on their child’s development, and they identified themselves as biological, foster, adoptive parents or other relatives of children aged birth to five years, residing in Nebraska and throughout the United States (referred to as “parents”).

We collected data on *N = *5,001 children in three phases (see Table 1) between October 1, 2020 and July 31, 2023. In the first phase (October 2020- February 2021), we obtained n *= *977 parent responses in the Lincoln and Omaha, Nebraska, USA metropolitan area to be used as pilot data; we followed up with n = 70 willing Phase 1 participants approximately 16 months (range: 12–24 months) following their initial survey response. In Phase 2 (August 2022- March 2023), we collected validity evidence from n = 2,428 parents throughout Nebraska, USA. Lastly, in Phase 3 (June-July 2023), we obtained item response data from n *= *1,596 parents throughout the United States through a limited and opportunistic partnership with Stanford Center for Early Childhood’s RAPID survey in which caregivers who were part of RAPID’s ongoing panel study were given the option to complete the KMT.

**Table 1:** Instrument development and validation study phases and collected measures.

	Phase 1		Phase 2	Phase 3
	Initial (N = 977)	Follow-Up (N = 70)	N = 2,428	N = 1,596
Study Year(s)	2020−21	2022	2022	2023
Location	Lincoln & Omaha, NE USA	Lincoln & Omaha, NE, USA	NE, USA (statewide)	United States (national)
	**Collected Measures**			
Child’s Race & Ethnicity	✓	–	✓	✓
Child’s Sex	✓	✓	✓	–
Family Income	✓	–	✓	✓
Caregiver Education	✓	–	✓	✓
PHQ- & GAD-2	✓^1^	–	✓	✓
Kidsights	✓	✓	✓	✓
D-score	✓	–	✓	–
CREDI	✓	–	✓	–
ECDI-2023 Score	✓	–	✓	–
HRTL	✓	–	✓	–
GSED-Psychosocial	✓	–	✓	✓
Bayley’s IV	–	✓	–	–
Woodcock-Johnson	–	✓	–	–

CREDI – Caregiver Reported Early Development Instrument; ECDI – Early Childhood Development Instrument; GAD – General Anxiety Disorder; GSED – Global Scales of Early Development; HRTL – Healthy and Ready to Learn; NE – Nebraska; PHQ – Patient Health Questionnaire; USA – United States of America.

^1^Random half-sample of caregivers.

Table 2 reports demographic information on participants with reference to the US national population estimates from the NSCH. Apart from the follow-up sample in Phase 1, we pooled the data in all three phases to calibrate the scale and assess validity evidence. In this pooled sample, 59.7% of the children whose parents reported on their development were identified as white, non-Hispanic compared to the national estimate of compared to the national estimate of 41.9%, and the mean age was 35.5 months. Data on the children’s sex was collected in Phase 1 and Phase 2, but child’s sex was opted not to be collected by the RAPID in Phase 3. In total across Phases 1 and 2, 53.3% of parents reported their child as male, and 97.6% of respondents reported that they were the biological, foster, or adoptive parent of the child.

**Table 2:** Sample demographics and family characteristics.

	Phase 1 (initial), Phase 2, & Phase 3		Phase 1 (follow-up)		US Population3	
	N = 5,001	%	N = 70	%	N = 36.61 (x106)	%
	Child Characteristics					
Sex^1^						
Female	1595	46.7	40	57.1	5.75	48.9
Male	1818	53.3	30	42.9	6.02	51.1
Age						
0–11 mo.	723	14.5	17	24.3	1.85	15.7
12–23 mo.	828	16.6	21	30	1.94	16.5
24–35 mo.	934	18.7	22	31.4	1.99	16.9
36–47 mo.	990	19.8	10	14.3	2.02	17.1
48–59 mo.	919	18.4	–	–	2.04	17.3
60–71 mo.	607	12.1	–	–	1.94	16.5
Race/Ethnicity						
White, non-Hispanic	2987	59.7	41	58.6	6.11	51.9
Black, non-Hispanic	453	9.1	6	8.6	1.40	11.9
Other/Two or more, non-Hisp.	672	13.4	8	11.4	1.42	12.1
Hispanic	889	17.8	15	21.4	2.85	24.2
						
	Caregiver/Household Characteristics					
Educational Attainment						
Less than HS Diploma	163	3.3	0	0.0	4.60	12.7
HS Diploma (or equivalent)	664	13.3	8	11.8	5.76	15.9
Some College or AA/AS	1607	32.1	11	16.2	10.20	28.0
Bachelor’s Degree or Higher	2565	51.3	49	72.0	15.80	43.5
						
						
Median Household Income	4636	$76,800	70	$75,900	35.02	$69,700
						
Anxiety and Depress. Sxs^2^ (0-Not at all, 5-Everyday)						
Feeling nervous, anxious, or on edge	4501	M = .88	36	M = .61		
Feeling down, depressed not being able to stop or control worrying hopeless	4491	M = .56	36	M = .22		
Not being able to stop or control worrying	4502	M = .69	36	M = .31		
little interest or pleasure in doing things	4500	M = .56	36	M = .25		

1 Phase 1–2 only.

2 Administered to a random half-sample in Phase 1 and to all participants in phases 2 and 3.

3 Estimates obtained from the National Survey of Children’s Health [29].

In the Phase 1 follow-up sample, 58.6% of children were identified as white, non-Hispanic. All respondents (100%) identified as the biological, foster, or adoptive parent, and 42.9% of parents reported their child as male.

### Procedures

In all phases, data were collected using the Qualtrics [30] online survey platform (Qualtrics, Provo, UT). We recruited parents through healthcare and childcare providers, parenting support programs, and social media posts. Parents received a link to an online questionnaire including several questions on family demographics, development and health. Respondents could complete the survey using their mobile phone, tablet, or computer and took between 20–30 minutes to complete. We offered parents a gift card to complete the survey. All protocols were reviewed by the Institutional Review Board of University of Nebraska Medical Center and Research Ethics Review Committee of the World Health Organization (ID ERC.0003514). To obtain informed consent, parents were given a written description of the study at the start along with the option to end their participation at any point. Acknowledgment of providing informed consent was documented in the Qualtrics platform. As part of a screening protocol, we excluded observations if (a) metadata information resulted in a “likely fraudulent” score from the *rIP* package [30] and the IP Hub database (https://iphub.info/), (b) the caregiver failed to accurately confirm the child’s birthdate, and (c) whether scores were above or below 5SD on the CREDI or ECDI (see below for a description of each).

For the Phase 1 follow up, parents that agreed to be recontacted received an email with a link to a short survey that included information about the activities, specifically the in-person appointment needed for the child direct assessment and the additional survey and asked for information to help schedule the in-person appointment. The assessment team scheduled appointments based on the family’s preference for data and location. The child direct assessments, specifically the Bayley Scales of Infant and Toddler Development, Fourth Edition (Bayley-4) [31] for children up to 42 months or the Woodcock Johnson IV Early Cognitive and Academic Development (WJ IV ECAD) [32] for children from 43 to 72 months, were conducted at the university, in the home or at the child’s childcare center when approved by the childcare provider. Parents received a link to the online survey including the KMT immediately after the child direct assessment appointment.

### Measures

#### Kidsights measurement tool.

The KMT was constructed by first forming a candidate item bank by adopting items from four previously validated instruments each measuring normative aspects of children’s development (i.e., skills or behaviors that children acquire or exhibit as they age when undergoing healthy development) between 0–5 years. These four instruments included (1) the Global Scale of Early Development Short Form (GSED-SF) [33], (2) the Caregiver Reported Early Development Instruments Long Form (CREDI-LF) [34], (3) the Early Childhood Development Index (ECDI2030) [35], and (4) Healthy and Ready to Learn (HRTL) [22]. We included only items that measured normative aspects of children’s development (i.e., skills or positive behaviors that are acquired or manifest as children age under healthy development), and we excluded items from these instruments that measure constructs such as problem behaviors or other indicators of psychosocial difficulties.

This process resulted in a candidate item bank of 223 items with 79 items unique to the GSED-SF, 23 items unique to the CREDI-LF, 7 items unique to the ECDI2030, and 49 items unique to the HRTL. Of the 223 items, 49 items were shared across one or more of the four contributing instruments (42 items were common between GSED-SF and CREDI; 7 items were common between the GSED-SF, CREDI-LF, and ECDI2030). The 223 candidate items measure motor, cognition, language, and/or social/emotional constructs according to the published literature and existing documentation for the four instruments. Specifically, 71 items represented fine or gross motor development constructs [33,34] or physical development [19] Additionally, 82 represented cognitive or language development [33–35] or early learning skills [19]. Lastly, 70 items measure social/emotional development [19,33–35] including normative aspects of children’s self-regulation [19]. In total, 224 candidate items were then screened and selected based on their psychometric properties (see S1 Table for details). The result was a final set of 197 items spanning development from birth to age 5 years, children are only administered 39 age-appropriate items, on average. Item stems and responses options are available in the S3 Table.

#### Concurrent and predictive measures.

The Bayley-4 or the WJ IV ECAD were administered to the Phase 1 follow-up subsample (N = 70). Specifically, the WJ IV ECAD was used for children from 43−60 months at follow-up (n = 33), and the Bayley-4 was used for children up to 42 months (n = 37).

***Bayley-4***. The Bayley-4 is validated to measure child development up to 42 months. The instrument is divided into items that capture development in the cognitive, language, motor, social/emotional and adaptive behavior domains through direct administration of activities, observation of the child, and questions to the caregiver [31]. Scores are provided at domain level and the subtest level. For the Bayley-4 training, assessors were required to complete a 12-hour online training hosted by the measure publisher. After completing the training, assessors submitted video recordings of administrations of the Bayley-4 or scheduled in-person observations with the research team’s Bayley-4 supervisor. The measure supervisor has several years of experience administering the Bayley Scales of Infant Development and evaluated each assessor for correct administration of item and scoring in order to certify each assessor as reliable on the Bayley-4.

***WJ IV ECAD***. The WJ IV ECAD is considered a measure of intellectual ability, academic skills and language, specifically oral expression for children 30 months to 6 years old [32]. The results of the WJ IV ECAD administrations result in a General Intellectual Ability Score, an Expressive Language Score, and scores by each test [19]. Although there are 10 tests in the WJ IV ECAD, only 7 of the tests were administered for the study. Assessors followed a similar training and reliability process for the WJ IV ECAD as for the Bayley-4. They reviewed the WJ IV ECAD kit materials and submitted video of the administration of the WJ IV ECAD. The research team’s WJ-ECAD supervisor has experience administering the measure and reviewed the recording for correct administration and scoring before certifying the assessors as reliable on the measure.

**Caregiver-reported instruments:** The candidate KMT item pool included all items from the GSED-SF, CREDI-LF, ECDI2030, and the items from HRTL as described in Ghandour et al. [19]. As a result, in administering the KMT, we effectively administered these four caregiver instruments concurrently. Scores from the GSED-SF are termed “D-scores” [36] and calculated using the *dscore* R package [37]. The CREDI Long Form results in an overall score of child development as well as subscale scores of motor, cognition, language and social/emotional development. We calculated CREDI scores using the *credi* R package (https://github.com/marcus-waldman/credi). ECDI2030 scores were calculated using the UNICEF’s (2023) provided R syntax file. HRTL scores were calculated by replicating the four-factor solution reported in Ghandour et al. [19] using the *lavaan* R package [38] and extracting factor scores. The four factors include a physical/motor factor, an early learning factor, a social/emotional factor, and a self-regulation factor.

#### Family- and caregiver-level criterion measures.

**Socioeconomic measures:** In the 2020 survey, we asked parents to report their 2019 household income in United States Dollars (USD). Likewise, in the 2022 survey, we asked parents to report their 2021 household income. To make household income on the same scale across years, we adjusted for inflation by converting to 1999 USD.

Parents could select from nine options in reporting their educational attainment: 1) 8th grade or less; 2) 9th-12th grade; 3) No diploma; 4) High School Graduate or GED Completed 5) Completed a vocational, trade, or business school program 6) Some College Credit, but No Degree 7) Associate Degree (AA, AS); 8) Bachelor’s Degree (BA, BS, AB); 9) Master’s Degree (MA, MS, MSW, MBA); 10) Doctorate (PhD, EdD) or Professional Degree (MD, DDS, DVM, JD). In the present study, we collapsed this information into four categories including 1) no high school (HS) diploma; 2) HS diploma; 3) Some college or an Associate’s degree (i.e., AA/AS); 4) Bachelor’s degree (i.e., BA/BS); and 5) Master’s degree or higher. We dummy coded caregiver educational attainment using parents with only high school education as the reference group.

**Anxiety and depressive symptoms:** We administered the Patient Health Questionnaire 2-item (PHQ-2) [39] and the Generalized Anxiety Disorder 2-item (GAD-2) [40] to obtain caregiver self-reports of depressive and anxiety symptoms. Parents reported whether, over the last two weeks, they (1) had little pleasure or interest in doing things (i.e., indicator 1 of PHQ-2) and (2) were feeling down, depressed, and hopeless (i.e., indicator 2 of PHQ-2), (3) were feeling nervous, anxious, or on edge (i.e., indicator 1 of GAD-2), and (4) were not able to stop or control worrying (indicator 2 of GAD-2). Parents responded using a four-point Likert scale (i.e., “0-Not at all”; “1-Several days”; “2-More than half the days”; “3-Nearly every day”). For analysis, we created a depression and anxiety symptom total score by summing all four PHQ/GAD-2 items.

#### Child’s race and ethnicity.

Parents could select from up to 15 racial categories and one of five ethnicity categories. Racial category response options included: 1) American Indian or Alaska Native; 2) Asian Indian; 3) Black or African American; 4) Chinese; 5) Filipino; 6) Guamanian or Chamorro; 7) Japanese; 8) Korean; 9) Native Hawaiian; 10) other Asian; 11) other Pacific Islander; 12) Samoan; 13) Vietnamese; 14) White; or 15) Some other race. Ethnicity response options included 1) No, not of Hispanic, Latino, or Spanish origin; 2) Yes, Mexican, Mexican American, Chicano; 3) Yes, Puerto Rican; 4) Yes, Cuban; 5) Yes, another Hispanic, Latino, or Spanish origin. For analysis, we combined racial and ethnicity into four major categories. These included: 1) White, non-Hispanic, 2) Black or African American, non-Hispanic, 3) Other (including two or more races), non-Hispanic, and 4) Hispanic.

#### Scaling and scoring procedures.

We fit the graded-response IRT model in (1)-(2) to the polytomous data.


$$\begin{array}{c} \mathrm{Pr}\left(Y_{j}\geq k\right)={logit}^{-1}\left(\alpha_{j}\left(\theta_{i}-\delta_{jk}\right)\right),\;\;0<k\leq K_{j}\; \end{array}$$ (1)

where i indexes a child (with a latent score [i.e., ability] of $\theta_{i}$), j indexes an item (with $K_{j}$ response options), and *k* indexes one of the responses options for the item. Model parameters in (1)-(2) include $\alpha_{j}$ (the item discrimination value), $\delta_{jk}$ (the difficulty value associated with response option k for item j), and a vector of latent regression coefficients, γ. We fit the model using maximum marginal likelihood estimation (also referred to as full information maximum likelihood). Maximum likelihood estimators are gold standard approaches to treating missing data [41] All model fitting occurred using the *mirt* R package [42]. We calculated scores from the KMT (“Kidsights scores”) by summarizing the posterior distribution using the expected-a-posteriori (EAP) point estimate.

### Analytic plan

We followed the recommendations from the APA *Standards* in collecting validity evidence. Evidence came from analyzing (1) test content, (2) relations with other variables (including criterion variables and scores from concurrent instrument), (3) the sensitivity of conclusions to threats from measurement non-invariance, and (4) score precision as indicated by errors of measurement (i.e., reliability).

#### Evidence based on test content.

**Fig 1:**
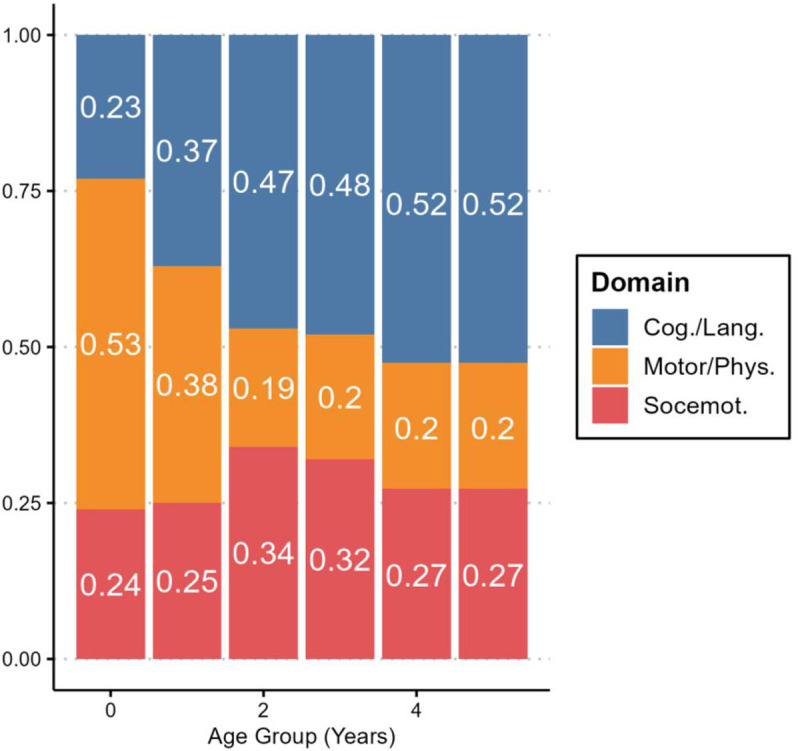
Item composition. Kidsights Measurement Tool domain representation by children’s age. Notes: Cog. – Cognition development, Lang. – Language development, Phys. – Physical development. Socemot. – Socioemotional development.

Using the domain assignments provided by the originating instruments (CREDI, GSED, HRTL and ECDI as described above), we designated items into one of three domains: (1) Motor/physical development, (2) Cognition or language development, or (3) Social/emotional development. To assess content coverage, we calculated the (average) domain composition of the administered items within yearly age categories. Evidence based on test content was evaluated by two subject matter experts to ensure adequate representation of items by developmental domain. See Fig 1 for a summary of item domains by age.

### Evidence based on other variables

In line with the *Standards*, we collected evidence that Kidsights scores correlate with other variables in the expected magnitude and direction. This includes: (a) convergent validity evidence with scores from concurrent instruments that measure equivalent (or highly similar) constructs as those directly intended to be measured by the KMT; (b) discriminant validity evidence with scores from concurrent measures measuring constructs *not* directly intended to be measured using the KMT; and (c) association of Kidsights scores with criterion variables known to be predictive of child development.

#### Convergent validity evidence.

Convergent validity evidence from calculating part correlations (i.e., correlations after adjusting for the child’s age) of Kidsights score with (a) the Bayley-4 Cognition, Receptive and Expressive Communication, and Gross and Fine Motor domain and subtest level scores for children 42 months and younger; and (b) the WJ IV ECAD General Intellectual Ability- Early Development scores, Expressive Language cluster scores, as well as the Verbal Analogies, Sentence Repetition, and Rapid Picture Naming test activities for children 43–60 months. Convergent validity evidence from caregiver-reported instruments came from part correlations with: (a) D-scores, (b) CREDI scores (overall scores, as well as motor, language, cognition, and social/emotional subscores), (c) ECDI2030 scores, and (d) motor/physical, early learning, and social/emotional factor scores from the HRTL. We are aware of no published ideal or minimum threshold for a correlation to establish convergent validity evidence. Because a correlation value of approximately r=.70 represents 50% of the variance explained, we used this value as an ideal threshold and a correlation of r=.50 as a minimum threshold (i.e., 25% of the variance explained).

#### Discriminant validity evidence.

Discriminant validity evidence from part correlations (i.e., correlations after controlling for children’s age) with scores from other instruments that reflect aspects of children’s behaviors which a not directly tied to normative aspects of children motor, cognitive, language, or social/emotional development. This included scores from the HRTL self-regulation factor and psychosocial problem scores from the GSED-PF. Because these scores reflect constructs not intended to be directly measured by the KMT, evidence comes from correlations smaller in magnitude (i.e., |r|<.50) and in the expected direction (i.e., negative part correlations with psychosocial problem scores, and positive correlations with self-regulation).

#### Predictive validity evidence.

We assessed predictive validity evidence by studying part correlations of Kidsights scores at Time 1 with Bayley-4 and WJ IV ECAD scores obtained 12–24 months (*M *= 16 months) later at Time 2. We took positive and statistically significant correlations as evidence that Kidsights scores predict future development and learning.

#### Criterion associations.

We fit multiple regression models to control for age and to assess bivariate associations with the following criterion variables: (a) family income (b) parent’s educational attainment (reference category: parents with a bachelor’s degree), (c) the child’s race and ethnicity (reference category: white, non-Hispanic children), and (d) parent’s depressive and anxiety symptoms. In fitting the regression models, family income was log-transformed to offset heavy right skew of that variable.

Criterion validity evidence includes positive associations between Kidsights scores and family income and greater levels of parent’s educational attainment. We expect to find a negative association with parent’s depressive and anxiety symptoms measured through a combined GAD/PHQ-2 total score. Lastly, we expect to find that historically disadvantaged groups (i.e., non-white and Hispanic children in the United States, children from lower levels of caregiver education, and those from lower family income households) will exhibit lower average Kidsights scores compared to peers of the same age.

As a result of the planned missingness in Phase 1 of the study for the GAD/PHQ-2 items (see above), missingness was most present in the GAD/PHQ-2 items resulting in 10.8% of the sample not having a GAD/PHQ-2 combined total score. Additionally, we found that 7.2% of parents did not provide an income. All other criterion variables contained no missingness. In response, we employed multiple imputation with 10 imputed datasets using the *mice* (van Buuren et al., 2015) R package to deal with missingness on the criterion variables. For all models, we evaluated the evidence from criterion associations by pooling results using Rubin’s rules, conducting pairwise t-tests, and interpreting the magnitude and substantive size of the coefficients.

#### Measurement noninvariance.

We evaluated whether items exhibited measurement noninvariance and conducted sensitivity analyses to determine if ignoring noninvariance threatened valid inferences. Using the alignment method [43], we tested for DIF across education, race and ethnicity groups, and between the Nebraska sample (i.e., Phase 1 [initial sample] and Phase 2 of the present study vs. Phase 3 [national sample]). We completed all invariance testing using Mplus Version 8.10 [44].

Because we are aware of no accepted standard that exists on the maximum percentage of items that exhibit noninvariance before valid inference is threatened, we conducted a sensitivity analysis in which we allowed all DIF items to be freely estimated when evaluating criterion associations with race and income, as well as group differences between the Nebraska (Phases 1 and 2) and the national samples. We compared associations and mean differences to the mean by ignoring DIF (i.e., assuming invariance across all items) to associations that allow for item parameters to be freely estimated for items exhibiting DIF. We employed generalized linear hypothesis tests to evaluate whether ignoring DIF results in significantly different conclusions about group differences.

#### Reliability and errors of measurement.

We assessed the precision of scores in two ways. First, we calculate the marginal reliability statistic ($r_{XX^{\prime }}$) proposed by Thissen and Wainer [45]  using the standard errors for the EAP estimates. Although the $r_{XX^{\prime }}$ is a valid measure of reliability, as a marginal statistic it overestimates the reliability when a child’s score is only to be compared to scores from their peers. To evaluate the precision of scores at conditional on a child’s age, we fit a generalized additive model for location scale and shape [46] to estimate age-conditional variances in EAP scores. Using the within age variance of EAP scores and the conditional standard error of measurement (CSEM), we then calculated the expected conditional reliability value ($r_{XX^{\prime }|{AGE}_{i}}$) for each child,


$$\begin{array}{c} r_{XX^{\prime }|{AGE}_{i}}=1-\frac{{C\hat{SE}M}_{i}^{2}}{V\hat{a}r\left\{{\hat{\theta }}^{EAP}|{AGE}_{i}\right\}},\# \end{array}$$ (2)

where ${\hat{\theta }}^{EAP}$ is the EAP score. We evaluated the average $r_{XX^{\prime }|{AGE}_{i}}$ values calculated in the previous step, in addition to evaluating the expected reliability $r_{XX^{\prime }|{AGE}_{i}}$ at each age. We used $r_{XX^{\prime }|{AGE}_{i}}=\mathrm{.80}$ as a cutoff for the minimal reliability at each age. Such a reliability value may be below traditional guidelines for individual assessments (i.e., when the conclusions are drawn regarding individuals). However, population measures like the KMT are intended to produce statistics that aggregate across individual scores, thereby washing out measurement error across individual score.

## Results

Demographics information in our sample is reported in Table 2. In total, 53.3% of children in the sample were identified as male. Moreover, majority of sampled children were identified as White, non-Hispanic (59.7%) from a parent reporting have attained a Bachelor’s degree or higher (51.3%).

### Evidence based on test content

Motor and physical development represented most items (53%) assigned in the first year of life, but these items represent only approximately 20% of administered items for children aged 2 years and older. In contrast, items identified as measuring cognitive or language development represent less than a quarter (23%) of items assigned to newborns, but these items represent a majority beginning at age 3 (Max = 52%). The percentage of items reflecting social/emotional development varies between 24% (for newborns) and 34% (for two-year-olds) (see Fig 1).

### Evidence based on other variables

#### Convergent validity evidence.

Part correlations of Kidsights scores with Bayley-4 and the WJ IV ECAD scores administered concurrently (i.e., each administered in the Phase 1 follow-up) are presented in Tables 2 & 3, respectively. With the Bayley-4, correlation coefficients were greater or equal to than the minimally acceptable threshold or r=.50(see Table 3). Kidsights scores correlated with Bayley-4 Expressive Communication scores and correlated with Baley-4 Fine Motor scores. For the WJ IV ECAD, the correlation between the Kidsights scores and the WJ IV ECAD scores for General Intellectual Ability- Early Development scores, Sentence Repetition scores, Expressive Language, Verbal Analogies, and Rapid Picture Naming test activities all met the minimally acceptable threshold (see Table 4). However, the part correlation between Kidsights scores with the WJ IV ECAD Picture Vocabulary scores were below the minimally acceptable threshold. Kidsights scores were not significantly correlated with Memory for Names, Sound Blending and Visual Closure scores from the WJ IV ECAD.

**Table 3:** Part correlations with scores from Bayley-4 administered at Time 2 (n = 37).

	(1)	(2)	(3)	(4)	(5)	(6)
1. Time 1 Kidsights	1.00					
					
					
2. Time 2 Kidsights	.66***	1.00				
	(.10)					
						
3. Cognition	.74***	.59***	1.00			
	(.07)	(.12)				
						
4. Receptive Communication	.64***	.56***	.95***	1.00		
	(.09)	(.11)	(.01)			
						
5. Expressive Communication	.77***	.63***	.92***	.90***	1.00	
	(.06)	(.10)	(.02)	(.02)		
						
6. Fine Motor	.73***	.50***	.94***	.90***	.90***	1.00
	(.08)	(.13)	(.01)	(.02)	(.02)	
						
7. Gross Motor	.75***	.57***	.87***	.79***	.83***	.87***
	(.07)	(.12)	(.03)	(.05)	(.04)	(.03)

Note: *** *p < .001*

**Table 4:** Part correlations with scores from WJ IV ECAD administered at Time 2 (n = 33).

	(1)	(2)	(3)	(4)	(5)	(6)	(7)	(8)	(9)	(10)
1. Time 1 Kidsights	1.00									
									
2. Time 2 Kidsights	.86***	1.00								
	(.04)									
3. GIA – Early Development	.66***	.70***	1.00							
	(.09)	(.08)								
4. Expressive Language	.59***	.66***	.92***	1.00						
	(.11)	(.09)	(.02)							
5. Memory for Names	.28	.24	.34*	.30	1.00					
	(.23)	(.22)	(.15)	(.16)						
6. Sound Blending	.37	.22	.38**	.17	−.02	1.00				
	(.21)	(.21)	(.14)	(.16)	(.18)					
7. Picture Vocabulary	.46**	.48***	.75***	.87***	.20	.08	1.00			
	(.16)	(.14)	(.04)	(.01)	(.16)	(.17)				
8. Verbal Analogies	.56***	.54***	.81***	.68***	−.01	.30	.57***	1.00		
	(.13)	(.12)	(.05)	(.08)	(.17)	(.16)	(.10)			
9. Visual Closure	−.08	.03	.24	.21	.28	−.15	.27	.06		
	(.33)	(.32)	(.22)	(.23)	(.20)	(.19)	(.21)	(.21)		
10. Sentence Repetition	.61***	.70*	.92***	.97***	.32*	.19	.73***	.67***	.14	
	(.11)	(.08)	(.02)	(.00)	(.16)	(.17)	(.02)	(.08)	(.23)	
11. Rapid Picture Naming	.54***	.63***	.82***	.68***	.29	.25	.42***	.60***	.05	.75***
	(.14)	(.11)	(.05)	(.08)	(.16)	(.17)	(.11)	(.10)	(.22)	(.06)

***p < .001, **p < .01, *p < .05

Table 4 provides convergent validity with scores derived from previously validated caregiver measures. Kidsights scores were highly correlated with D-scores and CREDI-LF Overall scores. Correlation coefficients were also greater than the ideal threshold value (i.e., r≥.70) for all CREDI subscores, ECDI2030 scores, and NOM early learning factor scores. Correlation coefficients were found to be greater than the minimally acceptable threshold value of *r *= .50 for HRTL physical development factor scores. Only the correlation with HRTL social/emotional factor scores were found to below the minimum threshold value we set.

#### Discriminant validity evidence.

As can be seen in Table 5, part correlations of Kidsights scores with HRTL self-regulation scores (*r *= .38, *p* *< .*001) and GSED-PF psychosocial problems scores (*r *= −.20, *p* *< *.001) provide supportive evidence of discriminant validity. The magnitude of each correlation was less than the magnitudes of the correlations used as evidence of convergent validity evidence. These correlations were also below the maximum threshold value (|r|<.50) and in the expected directions (i.e., a positive correlation with HRTL self-regulation scores and a negative correlation with GSED-PF psychosocial problem scores).

**Table 5:** Part correlations with CREDI and HRTL instruments (N = 5,001).

			(1)	(2)	(3)	(4)	(5)	(6)	(7)	(8)	(9)	(10)	(11)	(12)
	1.	Kidsights	1.00											
														
	2.	D-scores	.93***	1.00										
			(.01)											
														
CREDI	3.	Overall	.89***	.91***	1.00									
			(.01)	(.01)										
													
	4.	Motor	.82***	.86***	.87***	1.00								
			(.01)	(.01)	(.01)									
													
	5.	Cognition	.86***	.86***	.92***	.91***	1.00							
			(.01)	(.01)	(.01)	(.01)								
													
	6.	Language	.85***	.87***	.94***	.78***	.87***	1.00						
			(.01)	(.01)	(.01)	(.01)	(.01)							
													
	7.	Soc./Emot.	.82***	.81***	.87***	.85***	.97***	.80***	1.00					
			(.01)	(.01)	(.01)	(.01)	(.01)	(.01)						
														
	8.	ECDI2030	.75***	.70***	.71***	.67***	.71***	.76***	.66***	1.00				
			(.01)	(.02)	(.02)	(.02)	(.02)	(.02)	(.02)					
														
HRTL	9.	Physical	.62***	.52***	.45***	.49***	.44***	.43***	.41***	.48***	1.00			
			(.02)	(.03)	(.05)	(.05)	(.04)	(.03)	(.04)	(.02)				
													
	10.	Early Learning	.77***	.63***	.62***	.56***	.62***	.59***	.59***	.56***	.43***	1.00		
			(.01)	(.02)	(.03)	(.03)	(.03)	(.02)	(.03)	(.02)	(.02)			
													
	11.	Soc./Emot.	.39***	.37***	.42***	.39***	.39***	.34***	.38***	.30***	.16***	.21***	1.00	
			(.02)	(.03)	(.04)	(.04)	(.04)	(.04)	(.04)	(.03)	(.03)	(.03)		
													
	12.	Self Reg.	.38***	.33***	.39***	.33***	.37***	.34***	.35***	.24***	.16***	.27***	.36***	1.00
			(.02)	(.03)	(.04)	(.04)	(.04)	(.03)	(.04)	(.03)	(.03)	(.03)	(.02)	
														
	13.	GSED PF	−.20***	−.17***	−.14***	−.15***	−.18***	−.16***	−.18***	−.16***	−.12***	−.14***	−.43***	−.24***
			(.02)	(.02)	(.02)	(.02)	(.02)	(.02)	(.02)	(.02)	(.02)	(.02)	(.02)	(.02)
														

***p < .001, **p < .01, *p < .05.

#### Predictive validity evidence.

Correlations with Kidsights scores initially at Phase 1 and Bayley-4 or WJ IV ECAD scores at follow-up obtained 12−18 months later provide predictive validity evidence (see Tables 2,3). Kidsights scores were found to predict all Bayley-4 scores at Time 2; each correlation coefficient was positive, significant, and substantively large. Similarly, Kidsights scores predicted WJ IV ECAD GIA Early Development scores, Sentence Repetition scores, Expressive Language scores, Verbal Analogies scores, and Picture Vocabulary scores. However, we failed to find that Kidsights scores predicted WJ IV ECAD Memory for Names scores, Sound Blending scores, or Visual Closure scores.

#### Criterion associations.

Our results found evidence of associations between KMT and contextual factors, documenting early disparities. As expected, Kidsights scores were positively associated with family income (Est. = 0.040, SE = 0.007, *p < *.001, ES = .09). Moreover, compared to peers from parent’s reporting a bachelor’s degree or higher, children from parents with less educational attainment exhibited lower average Kidsights scores. (No high school diploma: Est. = −0.340, SE = .092, *p *< .001, ES = −0.34; High school diploma: Est. = −0.268, SE = .053, *p *< .001, ES = −0.27; Some college for associate’s degree: Est. = −0.081, SE = 0.039, *p *< .001, ES = .08). Children from parent’s were with a master’s degree or higher exhibited greater average scores in the sample compared children from parents with a bachelor’s degree, but this finding was not statistically significant (Est. = 0.051, SE = .039, *p *< .001, ES = 0.05).

Kidsights scores were negatively associated with parent’s anxiety and depressive symptoms (Est. = −0.298, SE = 0.039, *p* *< *.001, ES = −0.128), and non-white or Hispanic children exhibited lower average Kidsights scores compared to their white, non-Hispanic peers (Black or African-American, non-Hispanic: Est. = −0.137, SE = 0.058, *p* = .019, ES = −0.137; Other, non-Hispanic: Est. = −0.113, SE = 0.048, *p* = .018, ES = − 0.113; Hispanic: Est. = −0.237, SE = 0.042, *p* *< *.001, ES = −0.237)

#### Measurement noninvariance.

Kidsights scores met acceptable standards of to assert measurement invariance. Only 8 items (4.1%) exhibited DIF across education groups, 15 items exhibited DIF (7.1%) across racial and ethnicity groups, and 4 items (2.0%) exhibited DIF between the Nebraska sample (i.e., Phase 1 and 2 of the study) and nationally (i.e., Phase 3) sample (see the S2 Table on individual items). We found no evidence that allowing for approximate measurement invariance by allowing DIF items to be freely estimated across groups resulted in different conclusions about group differences (Education: $\chi^{2}=\mathrm{.341}$, df = 4, *p *= .987; Race and ethnicity: $\chi^{2}=\mathrm{.163}$, df = 3, *p *= .983; Geography: $\chi^{2}=\mathrm{.278}$, df = 1, *p *= .598)

#### Reliability and errors of measurement.

**Fig 2:**
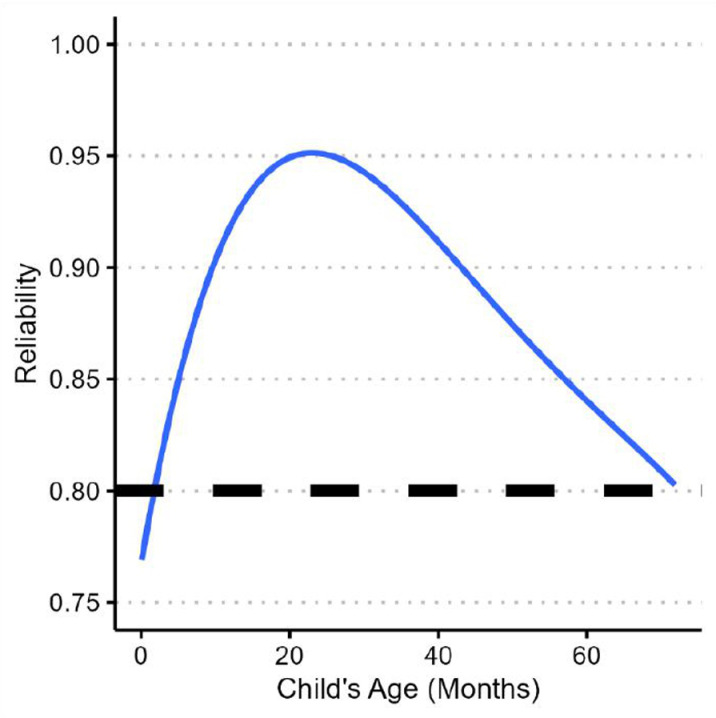
Reliability. Marginal reliability ($r_{XX^{\prime }}$of scores at child’s age (N = 5,001).

Reliability and errors of measurement estimate provide supportive evidence that measurement error does not unduly threaten population-level inferences. As expected, the marginal reliability was found to be very strong, $r_{XX^{\prime }}=\mathrm{.89}$. Controlling for variation due to children’s age, we found an average conditional reliability estimate of ${\overset{―}{r}}_{XX^{\prime }|AGE}=\mathrm{.89}$. Moreover, the expected conditional reliability was found to be greater than the minimum threshold value of .80, except for newborns for which the weakest reliability was approximately .77 at birth (see Fig 2).

## Discussion

Population-based measurement of early child development has the potential to generate new insight into the emergence of disparities in the earliest years of life. Because tracking and addressing early disparities is a cornerstone of effective public health systems, such data are critical. However, documenting these disparities requires feasible and reliable measurement tools designed to detect group-level differences in child development. This study is important because it introduces a new measure that can reliably and feasibly measure child development at the population level through an online parent-report survey. Our study documents multiple types of validity for the KMT and reports on its ability to detect population-level disparities. We demonstrate its feasibility for use at scale and document how KMT addresses the psychometric requirements detailed by the APA *Standards* for its intended use as a population-based measurement tool. Further, our findings indicate disparities in child development due to socio-economic status and caregiver mental health. Below we outline our findings in greater depth.

First, we found that KMT’s results address key aspects of validity. KMT’s test content demonstrates concordance with key domains of child development including cognitive, language, motor and social/emotional development. Although the proportion of items representing different domains varies by age, overall, the balance of domains represents relevant domains at each age, which was our goal. The process of selecting items was aided considerably by the reliance on existing scales of child development including the CREDI, GSED, and HRTL, each of which was developed through careful identification of developmental milestones in early childhood [19,24,25].

We found that Kidsights scores showed expected predictive and concurrent associations with scores from both direct observations and other parent-report measures designed for use at the population level, the CREDI, HRTL and ECDI. Beginning with the sample of 70 children assessed at two time points, we found evidence of predictive validity. Kidsights scores strongly predicted all Bayley-4 subscores observed 12−18 months later, meeting or exceeding the minimum acceptable threshold for association. Kidsights scores were also related to several of the WJ IV ECAD scores obtained 12−18 months after initial scores were collected. We found similar evidence of convergent validity when the KMT was administered concurrently with the Bayley-4 and the WJ IV ECAD, as well as associations with previously validated parent reported measures (i.e., GSED-SF, CREDI, and HRTL). While associations were largely confirmed by our data, we did not find that all subscales of the WJ IV ECAD were associated with Kidsights scores from a concurrent administration of the KMT. Specifically, the WJ IV ECAD subscales of memory for names, sound blending, and visual closure did not show concurrent associations with KMT scores, while picture vocabulary, verbal analogies, sentence repetition and rapid picture naming did. While these subscales are intended to index general intellectual ability [32], it is not clear from our study why some were related to the KMT and some were not, although some of the subscales were very difficult for children in our sample so the lack of positive association may have been due to overall low scores. Future work with KMT and indices of academic achievement, including following children later into the primary school years, may help clarify these findings. At the same time, the KMT is intended to be a holistic measure of child development rather than indexing specific skills, and thus may show greater sensitivity to some aspects of later academic achievement than others.

Importantly, our study offers insight on group-level early disparities in child development. Our results provide evidence towards criterion validity by demonstrating sensitivity to factors previously documented to influence child development and documenting early disparities in child development. Children whose parents reported greater depressive and anxious symptomology had lower Kidsights scores, all of which is consistent with existing research on threats to early child [47–49]. We also found positive associations between caregiver education and KMT, again aligned with existing findings on sources of disparity in child development demonstrating that parental education is associated with more positive developmental outcomes [50,51]. Documentation of these impacts across a large, diverse population of parents, and using a relatively brief and feasible tool, creates an opportunity to respond to government calls for more extensive monitoring of child development at the population level, as outlined above.

Our results also identify areas for further inquiry. We found smaller concurrent associations between Kidsights scores and HRTL subscales focused on social/emotional and self-regulatory development than with the HRTL early learning and physical development subscales. While the KMT contains indicators of social/emotional development, the majority of items are focused on cognitive, language and physical development. Further, HRTL social/emotional and self-regulatory subscales include indicators of problem behaviors as well as normative development, whereas the KMTl was focused exclusively on indexing social/emotional and regulatory competence. Going forward, we will continue to test KMTl’s sensitivity to early social and emotional development, including through the development of a new parent-report tool focused specifically on manifestations of early psychosocial stress [52].

There are limitations to our study that reflect challenges to widespread collection of population-level data on child development. Collecting a fully representative sample of young children requires random sampling, which we were not able to do. While our sample was aligned with the underlying population on key indicators such as overall income, parents in our sample, for example, reported less positive child health status and fewer child ACEs. To fulfill the vision of population-level tracking of early child development outlined by the Centers for Disease Control and others, investments in state and community infrastructure to obtain representative samples of parents is needed, as well as a concerted effort to expand data collection to include children birth to age three in the National Survey of Children’s Health.

As one step forward towards this long-term vision, our results offer evidence that KMT is a reliable and valid tool to measure holistic child development at the population level for children birth to age five. Identifying and tracking early disparities has significance for ensuring lifelong health and wellbeing, particularly for populations of young children who are vulnerable to high levels of stress due to the quality of their environments. When it becomes available soon as an open-source tool, the KMT will open the door to more extensive population-level monitoring of young children’s development beginning in the earliest years of life within large-scale programs or at the community, state or national levels, when addressing disparities is most cost effective.

## Supporting information

S1 File Validation of the Kidsights Measurement Tool: A Parent-Reported Instrument to Track Children’s Development at the Population Level. (DOCX)
