# Supplementary material for: Validation of the Kidsights Measurement Tool: A parent-reported instrument to track children’s development at the population level
Source: PLoS One. 2025 Jun 26;20(6):e0324082. doi: 10.1371/journal.pone.0324082 (PMC12200676; doi:10.1371/journal.pone.0324082)
Supplement: S1 File — (DOCX) [file pone.0324082.s001.docx]

Supplemental Materials

for

Validation of the Kidsights Measurement Tool: A Parent-Reported Instrument to Track Children’s Development at the Population Level

# Table of Contents

[Table of Contents 1](#_Toc199230374)

[Item Screening 2](#_Toc199230375)

[S1 Table. Items removed, corresponding domain, and reason for removal 2](#_Toc199230376)

[Item-level Information and Statistics 4](#_Toc199230377)

[S2 Table. Item parameter table (N = 5,001) 4](#_Toc199230378)

[Kidsights Measurement Tool 18](#_Toc199230379)

[S3 Table. Kidsights Measurement Tool 18](#_Toc199230380)

# Item Screening

We screened the candidate set of 224 items based on whether the items exhibited:

1. Positive age gradient, as predicted by development theory.
2. Sufficient item variability for newborn items beginning at birth to discriminate scores, as indicated by
   1. Predicted endorsement rates less than .90, and
   2. Item location outside the range of person locations, indicating minimal discrimination.
3. Substantial item misfit as indicated by RMSEA > .080

In total, 27 items failed to meet the screening criteria and were removed from consideration (see Supplementary Table 1 for details).

| S1 Table. Items removed, corresponding domain, and reason for removal. | | | |
| --- | --- | --- | --- |
|  | Item | Domain | Reason |
| R1 | When lying on his/her back, does your child move his/her arms and legs? | Motor/Phys. | Predicted item endorsement > .90 at 0 months. |
| R2 | When the child is upset, does he/she calm down quickly when you soothe or hold him/her? | Soc. Emot. | Predicted item endorsement > .90 at 0 months. |
| R3 | Does the child quickly calm down when he/she doesn't get what he/she wants? | Soc. Emot. | Predicted item endorsement > .90 at 0 months. |
| R4 | Does the child ask you for help using signs or words when he/she cannot do something on his/her own (e.g., to reach an object up high)? | Soc. Emot. | Predicted item endorsement > .90 at 0 months. |
| R5 | Does the child watch what other children do and try to copy them? | Soc. Emot. | Predicted item endorsement > .90 at 0 months. |
| R6 | Does the child stop at least briefly when told “no” or “stop that? | Soc. Emot. | Predicted item endorsement > .90 at 0 months. |
| R7 | Does the child show curiosity to learn new things (e.g., by asking questions or exploring a new area)? | Soc. Emot. | Predicted item endorsement > .90 at 0 months. |
| R8 | Does the child show sympathy or look concerned when others are hurt or sad? | Soc. Emot. | Predicted item endorsement > .90 at 0 months. |
| R9 | Is the child kind to younger children (e.g., speaks to them nicely and touches them gently)? | Soc. Emot. | Predicted item endorsement > .90 at 0 months. |
| R10 | Does your child cry when he/she is hungry, wet, tired, or wants to be held? | Cog./Lang. | Predicted item endorsement > .90 at 0 months. |
| R11 | Does your child look at and focus on objects in front of him/her? | Cog./Lang. | Predicted item endorsement > .90 at 0 months. |
| R12 | Does your child grasp your finger if you touch his/her hand? | Motor/Phys. | Predicted item endorsement > .90 at 0 months. |
| R13 | Can this child make a tower of three or more blocks? | Motor/Phys. | Predicted item endorsement > .90 at 0 months. |
| R14 | Can this child kick a ball? | Motor/Phys. | Predicted item endorsement > .90 at 0 months. |
| R15 | Does your child hold his/her hands in fists all the time? | Soc. Emot. | Non-positive association with age. |
| R16 | Does the child usually put objects or toys back where they belong after using them? | Soc. Emot. | Non-positive association with age. |
| R17 | Does your child bring his/her hand to his/her mouth? | Motor/Phys. | Item location outside of range of person location. |
| R18 | Does your child make sounds other than crying? | Cog./Lang. | Item location outside of range of person location. |
| R19 | When you talk to your child, does he/she smile, make noise, or more arms, legs or trunk in response? | Cog./Lang. | Poor item fit (RMSEA = 0.21). |
| R20 | When you say a word, how often can this child come up with another word that starts with the same sound? | Cog./Lang. | Poor item fit (RMSEA = 0.11). |
| R21 | If you say the word “cat,” how often can this child tell you a word that rhymes with “cat”? | Cog./Lang. | Poor item fit (RMSEA = 0.11). |
| R22 | Can your child write his/her own name? | Cog./Lang. | Poor item fit (RMSEA = 0.11). |
| R23 | Can your child pull themselves up from the floor while holding onto something? For example, can they pull themselves up using a chair, a person, or some other object? | Motor/Phys. | Poor item fit (RMSEA = 0.10). |
| R24 | Can this child consistently write his or her first name, even if some of the letters aren't quite right or are backwards? | Cog./Lang. | Poor item fit (RMSEA = 0.10). |
| R25 | How often can this child correctly add two numbers, like 2 plus 3? | Cog./Lang. | Poor item fit (RMSEA = 0.09). |
| R26 | How many letters of the alphabet can this child recognize? | Cog./Lang. | Poor item fit (RMSEA = 0.08). |
| R27 | Can your child take several steps (3-5) forward without holding onto any person or object, even if they fall down immediately afterward? | Motor/Phys. | Poor item fit (RMSEA = 0.08). |
|  |  |  |  |

# Item-level Information and Statistics

| S2 Table. Item parameter table (N = 5,001). | | | | | | | | | | | | | |
| --- | --- | --- | --- | --- | --- | --- | --- | --- | --- | --- | --- | --- | --- |
|  |  |  |  | Differential Item Functioning | | |  | Item Parameter Estimates | | | | | |
|  | Item | Domain | RMSEA | Edu. | Race/Eth. | Geo. |  | $\alpha$ | $\delta_{1}$ | $\delta_{2}$ | $\delta_{3}$ | $\delta_{4}$ | $\delta_{5}$ |
| 1 | Does your child stop crying or calm down when you come to the room after being out of sight, or when you pick him or her up? | Soc. Emot. | 0.022 | - | - | - |  | 0.55 | -10.65 | - | - | - | - |
| 2 | Does your child look at a person when that person starts talking or making noise? | Cog./Lang. | 0.025 | - | X | - |  | 0.755 | -9.905 | - | - | - | - |
| 3 | Does your child turn his/her head towards your voice or some noise? | Cog./Lang. | 0.031 | - | X | - |  | 0.799 | -9.481 | - | - | - | - |
| 4 | Does your child sometimes suck his/her thumb or fingers? | Soc. Emot. | 0.018 | - | - | - |  | 0.472 | -9.265 | - | - | - | - |
| 5 | When your child is on his/her stomach, can he/she turn his/her head to the side? | Motor/Phys. | 0.024 | - | X | - |  | 0.775 | -9.231 | - | - | - | - |
| 6 | Does your child look at your face when you speak to him/her? | Soc. Emot. | 0.034 | - | X | - |  | 0.941 | -8.98 | - | - | - | - |
| 7 | Does your child try to move his/her head (or eyes) to follow an object or person? | Motor/Phys. | 0.071 | - | X | - |  | 1.557 | -8.517 | - | - | - | - |
| 8 | When your child is on his/her stomach, can he/she hold his/her head up off the ground? | Motor/Phys. | 0.022 | - | X | - |  | 0.751 | -8.231 | - | - | - | - |
| 9 | How often does this child demonstrate an interest in books by choosing a children's book and turning pages? | Cog./Lang. | 0.036 | X | X | - |  | 0.24 | -8.179 | -1.034 | 4.888 | 12.088 | - |
| 10 | Can your child hold his/her head steady for at least a few seconds, without it flopping to the side? | Motor/Phys. | 0.033 | - | - | - |  | 1.032 | -8.162 | - | - | - | - |
| 11 | Does your child smile? | Soc. Emot. | 0.063 | - | X | - |  | 1.379 | -8.154 | - | - | - | - |
| 12 | While your child is on his/her back, can he/she bring his/her hands together? | Motor/Phys. | 0.021 | - | - | - |  | 0.72 | -8.102 | - | - | - | - |
| 13 | Does your child make noise or gesture to get your attention? | Cog./Lang. | 0.023 | - | - | - |  | 0.749 | -7.728 | - | - | - | - |
| 14 | Does the child react differently according to the tone of your voice (e.g., smiles when you say something in a happy tone)? | Soc. Emot. | 0.029 | - | - | - |  | 0.345 | -7.424 | - | - | - | - |
| 15 | Does your child smile when you smile or talk with him/her? | Soc. Emot. | 0.107 | - | X | - |  | 2.39 | -7.169 | - | - | - | - |
| 16 | Does your child make sounds when LOOKING at toys or people (not crying)? | Cog./Lang. | 0.052 | - | X | - |  | 1.501 | -7.062 | - | - | - | - |
| 17 | When you are about to pick up your child, does he/she act happy or excited? | Soc. Emot. | 0.039 | - | - | - |  | 1.242 | -6.817 | - | - | - | - |
| 18 | Does your child grasp onto a small object (e.g., your finger, a spoon) when out in his/her hand? | Motor/Phys. | 0.027 | - | - | - |  | 0.875 | -6.696 | - | - | - | - |
| 19 | Does your child move excitedly, kick legs, move arms or trunk, or make coo noises when a known person enters the room or speaks to them? | Soc. Emot. | 0.102 | - | - | - |  | 1.251 | -6.407 | - | - | - | - |
| 20 | Does the child smile when others smile at him/her? | Soc. Emot. | 0.035 | - | X | - |  | 1.716 | -6.389 | - | - | - | - |
| 21 | Does your child recognize you or other family members (e.g., smile when they enter a room or move toward them)? | Cog./Lang. | 0.039 | - | - | - |  | 1.799 | -6.266 | - | - | - | - |
| 22 | Does your child smile or become excited when seeing someone familiar? | Soc. Emot. | 0.04 | - | - | - |  | 2.352 | -6.176 | - | - | - | - |
| 23 | When held in a sitting position, can the child hold his/her head steady and straight? | Motor/Phys. | 0.014 | - | - | - |  | 1.479 | -5.391 | - | - | - | - |
| 24 | If you play a game with your child, does he/she respond with interest? For example, if you play peek-a-boo, pat-a-cake, wave bye-bye, etc. does your child smile, widen their eyes, kick or move arms or vocalize? | Soc. Emot. | 0.023 | - | - | - |  | 1.362 | -5.277 | - | - | - | - |
| 25 | When he/she is on his/her tummy, can your child hold his/her head straight up, looking around for more than a few seconds? He/she can rest on his/her arms while doing this. | Motor/Phys. | 0.018 | - | - | - |  | 1.181 | -5.237 | - | - | - | - |
| 26 | Does your child laugh? | Cog./Lang. | 0.005 | - | - | - |  | 1.757 | -4.967 | - | - | - | - |
| 27 | Does the child quickly calm down when he/she doesn't get what he/she wants? | Soc. Emot. | 0.007 | - | - | - |  | 0.317 | -4.805 | - | - | - | - |
| 28 | Is your child interested when he/she sees other children playing? Does she or he watch, smile, or look excited? | Soc. Emot. | 0.021 | - | - | - |  | 1.249 | -4.78 | - | - | - | - |
| 29 | Does your child show interest in new objects that are put in front of him/her by reaching out for them? | Cog./Lang. | 0.004 | - | - | - |  | 1.993 | -4.648 | - | - | - | - |
| 30 | Child bounces back easily when things do not go his/her way. | Soc. Emot. | 0.026 | - | - | - |  | 0.319 | -4.618 | 3.626 | 6.566 | 14.436 | - |
| 31 | Can your child reach for AND HOLD an object, at least for a few seconds? | Motor/Phys. | 0.045 | - | - | - |  | 1.682 | -4.487 | - | - | - | - |
| 32 | When lying on his/her stomach, can your child hold his/her head and chest off the ground using only his/her hands and arms for support? | Motor/Phys. | 0.009 | - | - | - |  | 1.451 | -4.326 | - | - | - | - |
| 33 | When necessary, how often does this child listen to adults? | Soc. Emot. | 0.029 | - | - | - |  | 0.475 | -4.321 | 1.607 | 4.053 | 10.831 | - |
| 34 | Does your child make single sounds like "buh" or "duh" or "muh"? | Cog./Lang. | 0.004 | - | - | - |  | 1.06 | -4.175 | - | - | - | - |
| 35 | Can your child roll from his/her back to stomach or stomach to his/her side? | Motor/Phys. | 0.012 | - | - | - |  | 1.284 | -3.993 | - | - | - | - |
| 36 | Can your child roll from his/her back to stomach, or stomach to back, on his/her own? | Motor/Phys. | 0.008 | X | - | - |  | 1.337 | -3.833 | - | - | - | - |
| 37 | Does the child often show affection toward others (e.g., hugging parents, brothers, or sisters)? | Soc. Emot. | 0.018 | - | - | - |  | 0.527 | -3.781 | - | - | - | - |
| 38 | Can your child sit with support, either leaning against something (furniture or person) or by leaning forward on his or her hands? | Motor/Phys. | 0 | - | - | - |  | 1.612 | -3.767 | - | - | - | - |
| 39 | Does your child try to reach for objects that are in front of him/her by extending one or both arms? | Motor/Phys. | 0.006 | - | - | - |  | 2.487 | -3.663 | - | - | - | - |
| 40 | When lying on his/her back, does the child grab his/her feet? | Motor/Phys. | 0 | - | - | - |  | 1.657 | -3.584 | - | - | - | - |
| 41 | Can your child pick up a small object (e.g., a small toy or small stone) using just one hand? | Motor/Phys. | 0 | - | - | - |  | 2.409 | -3.379 | - | - | - | - |
| 42 | Does your child intentionally move or change his/her position to get objects that are out of reach? | Cog./Lang. | 0 | - | - | - |  | 2.566 | -3.232 | - | - | - | - |
| 43 | Does the child look for an object of interest when it is removed from sight or hidden from him/her (e.g., put under a cover, behind another object)? | Cog./Lang. | 0 | - | - | - |  | 1.562 | -3.099 | - | - | - | - |
| 44 | Can your child eat food from your fingers or off a spoon you hold? | Soc. Emot. | 0.019 | - | - | - |  | 2.453 | -3.025 | - | - | - | - |
| 45 | If an object falls to the ground out of view, does your child look for it? | Motor/Phys. | 0 | - | - | - |  | 2.028 | -2.877 | - | - | - | - |
| 46 | Does your child play by tapping an object on the ground or a table? | Cog./Lang. | 0 | - | - | - |  | 3.333 | -2.875 | - | - | - | - |
| 47 | When you put your child on the floor, can she lean on her hands while sitting? | Motor/Phys. | 0.014 | - | - | - |  | 2.079 | -2.803 | - | - | - | - |
| 48 | Even if your child is unable to do singing games, does he/she enjoy them and want to be a part of them? | Soc. Emot. | 0.005 | - | - | - |  | 0.743 | -2.68 | - | - | - | - |
| 49 | Can your child bang objects together, or bang an object on the table or on the ground? | Motor/Phys. | 0.014 | - | - | - |  | 2.726 | -2.648 | - | - | - | - |
| 50 | Can your child pass a small object from one hand to the other? | Motor/Phys. | 0 | - | - | - |  | 2.714 | -2.526 | - | - | - | - |
| 51 | Does your child make two similar sounds together like baba, mumu, pepe, didi (single consonant vowel combinations)? | Cog./Lang. | 0.014 | - | - | - |  | 1.663 | -2.353 | - | - | - | - |
| 52 | Can your child hold him/herself in a sitting position without help or support for longer than a few seconds? | Motor/Phys. | 0 | - | - | - |  | 3.543 | -2.251 | - | - | - | - |
| 53 | How often does this child play well with others? | Soc. Emot. | 0.03 | - | - | - |  | 0.556 | -2.174 | 1.584 | 3.152 | 7.91 | - |
| 54 | Can your child maintain a standing position while holding on to a person or object (e.g., wall or furniture)? | Motor/Phys. | 0.006 | - | - | - |  | 1.675 | -1.979 | - | - | - | - |
| 55 | How often does this child show concern when they see others are hurt or unhappy? | Soc. Emot. | 0.029 | - | - | - |  | 0.482 | -1.834 | 2.364 | 3.866 | 8.411 | - |
| 56 | Can your child pick up small bits of food and feed him/her-self using his/her hand? | Soc. Emot. | 0 | - | - | - |  | 2.456 | -1.742 | - | - | - | - |
| 57 | Can your child pick up a small object (e.g., a piece of food, small toy or small stone) with just his/her thumb and one finger? | Motor/Phys. | 0.019 | - | - | - |  | 1.675 | -1.66 | - | - | - | - |
| 58 | Does your child stop what he/she is doing when you say “Stop!” even if just for a second? | Cog./Lang. | 0.022 | - | - | - |  | 1.151 | -1.583 | - | - | - | - |
| 59 | Can your child pick up and drop a small object (e.g., a small toy or small stone) into a bucket or bowl while sitting? | Motor/Phys. | 0 | - | - | - |  | 2.855 | -1.264 | - | - | - | - |
| 60 | How often does child take turns during games or fun activities? | Soc. Emot. | 0.038 | - | - | - |  | 0.628 | -1.14 | 3.743 | 5.514 | 9.906 | - |
| 61 | When the child is upset, does he/she calm down quickly on his/her own? | Soc. Emot. | 0.036 | - | - | - |  | 0.146 | -0.784 | - | - | - | - |
| 62 | While holding onto furniture, does your child squat with control (without falling or flopping down)? | Motor/Phys. | 0.024 | - | - | - |  | 4.279 | -0.733 | - | - | - | - |
| 63 | While holding onto furniture, does your child bend down and pick up a small object from the floor and then return to a standing position? | Motor/Phys. | 0 | - | - | - |  | 4.128 | -0.675 | - | - | - | - |
| 64 | Can your child walk several steps while holding on to a person or object (e.g., wall or furniture)? | Motor/Phys. | 0.003 | - | - | - |  | 3.004 | -0.665 | - | - | - | - |
| 65 | How often does this child keep working at a task when things don’t work out? | Soc. Emot. | 0.034 | - | - | - |  | 0.515 | -0.665 | 4.653 | 7.149 | 12.428 | - |
| 66 | How often does this child keep working at a task after setbacks? | Soc. Emot. | 0.035 | - | - | - |  | 0.581 | -0.606 | 4.255 | 6.433 | 11.469 | - |
| 67 | Does the child imitate others' behaviors (e.g., washing hands or dishes)? | Soc. Emot. | 0.049 | X | - | - |  | 0.409 | -0.468 | - | - | - | - |
| 68 | Does your child share with others (e.g., food)? | Soc. Emot. | 0.012 | - | - | - |  | 0.961 | -0.379 | - | - | - | - |
| 69 | Can your child climb onto an object (rock, porch, step, chair, bed, low table, etc.)? | Motor/Phys. | 0.027 | - | - | - |  | 2.303 | -0.188 | - | - | - | - |
| 70 | Can your child stand up without holding onto anything, even if just for a few seconds? | Motor/Phys. | 0.025 | - | - | - |  | 2.857 | -0.183 | - | - | - | - |
| 71 | Does your child make a gesture to indicate “No” (e.g., shaking head)? | Cog./Lang. | 0.005 | - | - | - |  | 1.398 | -0.146 | - | - | - | - |
| 72 | Can your child make any light marks on paper or in dirt with a crayon or a stick? | Motor/Phys. | 0.014 | - | - | - |  | 1.938 | -0.086 | - | - | - | - |
| 73 | Can your child stack at least two objects on top of each other, such as bottle tops, blocks, stones, etc.? | Motor/Phys. | 0.005 | - | - | - |  | 1.684 | 0.147 | - | - | - | - |
| 74 | Can your child follow a simple spoken command or direction without you making a gesture? | Cog./Lang. | 0.013 | - | - | - |  | 1.951 | 0.182 | - | - | - | - |
| 75 | Can your child make a scribble on paper, or in dirt, in a back and forth manner? For example, can he or she move the pen or pencil or stick back and forth? | Motor/Phys. | 0.018 | - | - | - |  | 1.991 | 0.243 | - | - | - | - |
| 76 | Can your child maintain a standing position on his/her own, without holding on or receiving support? | Motor/Phys. | 0.024 | - | - | - |  | 3.722 | 0.246 | - | - | - | - |
| 77 | Does your child get along well with other children? | Soc. Emot. | 0.018 | X | - | X |  | 0.639 | 0.307 | - | - | - | - |
| 78 | Can your child greet people either by giving his/her hand or saying “hello”? | Soc. Emot. | 0.012 | - | - | - |  | 1.324 | 0.352 | - | - | - | - |
| 79 | Can your child bend down or squat to pick up an object from the floor and then stand up again, without help from a person or object? | Motor/Phys. | 0.041 | - | - | - |  | 3.889 | 0.374 | - | - | - | - |
| 80 | Can your child break off pieces of food and feed them to him/her-self? | Soc. Emot. | 0 | - | - | - |  | 1.083 | 0.465 | - | - | - | - |
| 81 | Can your child move around by walking, rather than by crawling on his hands and knees? | Motor/Phys. | 0.042 | - | - | - |  | 2.938 | 0.54 | - | - | - | - |
| 82 | Can your child stand up from sitting by himself and take several steps forward? | Motor/Phys. | 0.053 | - | - | - |  | 3.375 | 0.545 | - | - | - | - |
| 83 | While standing, can your child purposefully throw the ball and not just drop it? | Motor/Phys. | 0.036 | - | - | - |  | 1.601 | 0.591 | - | - | - | - |
| 84 | Can your child fetch something when asked? | Cog./Lang. | 0.019 | - | - | - |  | 1.899 | 0.676 | - | - | - | - |
| 85 | Does your child put his/her hands out to have them washed? | Soc. Emot. | 0 | - | - | - |  | 1.494 | 0.753 | - | - | - | - |
| 86 | Can the child sit or play on his/her own for at least 20 minutes? | Soc. Emot. | 0.012 | - | - | - |  | 0.628 | 0.919 | - | - | - | - |
| 87 | Can your child walk well, with coordination, without falling down often? With one foot in front of the other (rather than shifting weight side to side, stiff- legged)? | Motor/Phys. | 0.021 | - | - | - |  | 2.257 | 0.944 | - | - | - | - |
| 88 | Does the child usually follow rules and obey adults (e.g., go there or don't do that)? | Soc. Emot. | 0.011 | - | - | - |  | 0.583 | 0.96 | - | - | - | - |
| 89 | Can your child kick a ball or other round object forward using his/her foot? | Motor/Phys. | 0.014 | - | - | - |  | 1.836 | 1.064 | - | - | - | - |
| 90 | Can your child say five or more separate words (e.g., names like "Mama" or objects like "ball")? | Cog./Lang. | 0.019 | - | - | - |  | 1.787 | 1.107 | - | - | - | - |
| 91 | Can your child stack three or more small objects (e.g., blocks, cups, bottle caps) on top of each other? | Motor/Phys. | 0.009 | - | - | - |  | 1.99 | 1.11 | - | - | - | - |
| 92 | Can your child walk on an uneven surface (e.g., a bumpy or steep road) without falling? | Motor/Phys. | 0.02 | - | - | - |  | 1.458 | 1.353 | - | - | - | - |
| 93 | Does the child sometimes share things (e.g., food, toys) with others without being told? | Soc. Emot. | 0.015 | - | - | - |  | 0.836 | 1.445 | - | - | - | - |
| 94 | While standing, can your child kick a ball by swinging his/her leg forward? | Motor/Phys. | 0.019 | - | - | - |  | 2.009 | 1.472 | - | - | - | - |
| 95 | Can your child run well, without falling or bumping into objects? | Motor/Phys. | 0.023 | - | - | - |  | 1.958 | 1.573 | - | - | - | - |
| 96 | Does your child show independence (e.g., wants to pick their own clothes, choose their own activities, or tries to go outside alone)? | Soc. Emot. | 0.01 | - | - | - |  | 1.292 | 1.596 | - | - | - | - |
| 97 | Can your child identify at least seven objects? For example, when you ask 'where is the ball/spoon/cup/cloth/door/plate/bucket etc.' does your child look at or point to (or even name) the objects? | Cog./Lang. | 0 | - | - | - |  | 2.537 | 1.614 | - | - | - | - |
| 98 | Can the child tell you when he/she is tired or hungry? | Soc. Emot. | 0.013 | - | - | - |  | 1.137 | 1.636 | - | - | - | - |
| 99 | Can your child correctly name at least one family member other than mom and dad (e.g., name of brother, sister, aunt, uncle)? | Cog./Lang. | 0.023 | - | - | - |  | 1.759 | 1.66 | - | - | - | - |
| 100 | Does the child involve others in play (i.e., play interactive games with other children)? | Soc. Emot. | 0.007 | - | - | - |  | 0.949 | 1.676 | - | - | - | - |
| 101 | Can your child follow directions with more than one step? For example, "Go to the kitchen and bring me a spoon." | Cog./Lang. | 0.014 | - | - | - |  | 2.441 | 1.746 | - | - | - | - |
| 102 | Can your child drink from an open cup without help? | Motor/Phys. | 0 | - | - | - |  | 1.068 | 1.778 | - | - | - | - |
| 103 | Can your child name at least two body parts (e.g., arm, eye, or nose)? | Cog./Lang. | 0.011 | - | - | - |  | 2.483 | 1.933 | - | - | - | - |
| 104 | Can your child say ten or more words in addition to "Mama" and "Dada"? | Cog./Lang. | 0.023 | - | - | - |  | 2.529 | 1.94 | - | - | - | - |
| 105 | If you show your child an object he/she knows well (e.g., a cup or animal), can he/she consistently name it? | Cog./Lang. | 0.025 | - | - | - |  | 2.714 | 1.943 | - | - | - | - |
| 106 | Does the child listen to someone telling a story with interest? | Soc. Emot. | 0.006 | - | - | - |  | 1.068 | 1.975 | - | - | - | - |
| 107 | Can this child feed self with a spoon with little spilling? | Motor/Phys. | 0 | - | - | - |  | 1.108 | 1.999 | - | - | - | - |
| 108 | Can this child open doors by turning a doorknob or handle? | Motor/Phys. | 0.012 | - | - | - |  | 1.108 | 2.082 | - | - | - | - |
| 109 | Can your child remove an item of clothing (e.g., take off his/her shirt)? | Motor/Phys. | 0.015 | - | - | - |  | 1.173 | 2.084 | - | - | - | - |
| 110 | Can your child ask for something (e.g., food, water) by name when he/she wants it? | Cog./Lang. | 0.045 | - | - | - |  | 2.683 | 2.117 | - | - | - | - |
| 111 | How often can this child explain things they have seen or done so that you understand? | Cog./Lang. | 0.055 | - | - | - |  | 1.064 | 2.138 | 4.051 | 4.837 | 7.058 | - |
| 112 | Does your child help out around the house with simple chores, even if he/she doesn't do them well? | Soc. Emot. | 0.012 | - | - | - |  | 1.038 | 2.15 | - | - | - | - |
| 113 | Can this child throw a ball overhand? | Motor/Phys. | 0.014 | - | - | - |  | 0.89 | 2.159 | - | - | - | - |
| 114 | Can this child climb stairs with one foot on each stair? | Motor/Phys. | 0.025 | - | - | - |  | 0.732 | 2.228 | - | - | - | - |
| 115 | Does the child greet neighbors or other people he/she knows without being told (e.g., by saying hello or gesturing hello)? | Soc. Emot. | 0.01 | - | - | - |  | 0.753 | 2.239 | - | - | - | - |
| 116 | Does your child dry hands by herself/himself after you have washed them? | Motor/Phys. | 0.01 | - | - | - |  | 1.579 | 2.276 | - | - | - | - |
| 117 | When looking at pictures, if you say to your child "what is this?", can they say the name of the object that you point to? | Cog./Lang. | 0.027 | - | - | - |  | 3.253 | 2.321 | - | - | - | - |
| 118 | Can your child say 15 or more separate words (e.g., names like "Mama" or objects like "ball")? | Cog./Lang. | 0.027 | - | - | - |  | 2.454 | 2.419 | - | - | - | - |
| 119 | Can your child speak using short sentences of two words that go together (e.g., 'Mama go' or 'Dada eat')? | Cog./Lang. | 0.034 | - | - | - |  | 2.981 | 2.443 | - | - | - | - |
| 120 | Can the child sit still when asked to by an adult (e.g., for two minutes)? | Soc. Emot. | 0 | - | - | - |  | 0.908 | 2.453 | - | - | - | - |
| 121 | Does your child usually communicate with words what he/she wants in a way that is understandable to others? | Cog./Lang. | 0.021 | - | - | - |  | 1.854 | 2.478 | - | - | - | - |
| 122 | How high can this child count correctly? | Cog./Lang. | 0.063 | - | - | - |  | 1.398 | 2.515 | 3.721 | 5.017 | 5.631 | 6.7 |
| 123 | Can your child do an activity such as coloring without repeatedly asking for help or giving up too quickly? | Soc. Emot. | 0.032 | - | - | - |  | 0.772 | 2.542 | - | - | - | - |
| 124 | Can the child name at least one color (e.g., red, blue, yellow)? | Cog./Lang. | 0 | - | - | - |  | 2.374 | 2.542 | - | - | - | - |
| 125 | Can the child easily switch back and forth between activities (e.g., go back to a game after being interrupted)? | Soc. Emot. | 0 | X | - | - |  | 0.858 | 2.547 | - | - | - | - |
| 126 | Can your child jump with both feet leaving the ground? | Motor/Phys. | 0 | - | - | - |  | 1.781 | 2.578 | - | - | - | - |
| 127 | If asked to count objects, how high could this child count correctly? | Cog./Lang. | 0.071 | - | - | - |  | 1.466 | 2.583 | 3.947 | 5.27 | 5.912 | 6.882 |
| 128 | Does this child repeat or sing rhymes? | Cog./Lang. | 0.028 | - | - | - |  | 0.954 | 2.586 | - | - | - | - |
| 129 | Does your child show respect around elders? | Soc. Emot. | 0.018 | - | - | - |  | 1.058 | 2.594 | - | - | - | - |
| 130 | Can this child catch a large ball with both hands? | Motor/Phys. | 0.023 | - | - | - |  | 0.782 | 2.641 | - | - | - | - |
| 131 | Does the child usually finish an activity he/she enjoys (e.g., a game or book)? | Soc. Emot. | 0 | - | - | - |  | 0.965 | 2.704 | - | - | - | - |
| 132 | Can your child sing a short song or repeat parts of a rhyme from memory by him/herself? | Cog./Lang. | 0.012 | - | X | - |  | 2.152 | 2.817 | - | - | - | - |
| 133 | How often can this child recognize the beginning sound of a word? For example, the word “ball” starts with the “buh” sound? | Cog./Lang. | 0.066 | X | - | - |  | 0.884 | 2.82 | 5.369 | 6.103 | 8.081 | - |
| 134 | Can your child tell you or someone familiar his/her own name/nickname when asked to? | Cog./Lang. | 0.006 | - | X | - |  | 2.34 | 2.851 | - | - | - | - |
| 135 | Does your child ask about familiar people other than parents when they are not there, for example, “Where is Grandma?” | Soc. Emot. | 0.01 | - | - | - |  | 1.746 | 2.956 | - | - | - | - |
| 136 | Can the child concentrate on one task (e.g., playing with friends, eating meal) for 20 minutes? | Soc. Emot. | 0.013 | - | - | - |  | 0.727 | 2.959 | - | - | - | - |
| 137 | Can your child tell you when others are happy, angry, or sad? | Soc. Emot. | 0.011 | - | - | - |  | 1.877 | 2.968 | - | - | - | - |
| 138 | When using a pencil, can he or she use fingers to hold it? | Motor/Phys. | 0.034 | - | - | - |  | 0.905 | 2.994 | - | - | - | - |
| 139 | Can your child tell you when he/she is happy, angry, or sad? | Soc. Emot. | 0.013 | - | - | - |  | 1.898 | 3.026 | - | - | - | - |
| 140 | Can this child identify: a circle? | Cog./Lang. | 0.008 | - | - | - |  | 1.858 | 3.035 | - | - | - | - |
| 141 | Can this child sort objects by: color? | Cog./Lang. | 0.01 | - | - | X |  | 1.695 | 3.052 | - | - | - | - |
| 142 | If shown two balls, could this child point to the larger ball? | Cog./Lang. | 0 | - | - | - |  | 1.955 | 3.058 | - | - | - | - |
| 143 | Does your child know the difference between the words "big" and "small"?For example, if you ask, "Give me the big spoon" can your child understand which one to give if there are two different sizes? | Cog./Lang. | 0.019 | - | - | - |  | 2.352 | 3.062 | - | - | - | - |
| 144 | Is this child able to do the following: Understand “in,” “on,” and “under”? | Cog./Lang. | 0.022 | - | - | - |  | 1.179 | 3.065 | - | - | - | - |
| 145 | If you show your child two objects or people of a different size, can he/she tell you which one is the big one and which is the small one? | Cog./Lang. | 0.011 | - | - | - |  | 2.5 | 3.069 | - | - | - | - |
| 146 | Can your child speak using sentences of three or more words that go together (e.g., "I want water" or "The house is big")? | Cog./Lang. | 0.037 | - | - | - |  | 3.13 | 3.089 | - | - | - | - |
| 147 | Does the child ask about familiar people other than parents when they are not there (e.g., Where is the neighbor?)? | Soc. Emot. | 0 | - | - | - |  | 1.661 | 3.09 | - | - | - | - |
| 148 | Can your child stand on one foot WITHOUT any support for at least a few seconds? | Motor/Phys. | 0.009 | - | - | - |  | 1.307 | 3.113 | - | - | - | - |
| 149 | Does your child pronounce most of his/her words correctly? | Cog./Lang. | 0 | - | - | - |  | 1.51 | 3.115 | - | - | - | - |
| 150 | Can your child put on at least one piece of clothing by himself? | Soc. Emot. | 0.01 | - | - | - |  | 1.569 | 3.115 | - | - | - | - |
| 151 | Can this child jump forward at least six inches? | Motor/Phys. | 0.021 | - | - | - |  | 1.126 | 3.168 | - | - | - | - |
| 152 | Does your child know to keep quiet when the situation requires it? (e.g., at ceremonies, when someone is asleep) | Soc. Emot. | 0.012 | - | - | - |  | 0.809 | 3.182 | - | - | - | - |
| 153 | Does your child offer to help someone who seems to need help? | Soc. Emot. | 0.022 | X | - | - |  | 1.138 | 3.241 | - | - | - | - |
| 154 | Can your child say what he/she likes or dislikes (e.g., "I like sweets")? | Soc. Emot. | 0.023 | - | - | - |  | 3.196 | 3.247 | - | - | - | - |
| 155 | Can your child wash hands by him/herself? | Motor/Phys. | 0 | - | - | - |  | 1.75 | 3.25 | - | - | - | - |
| 156 | Can your child unscrew the lid from a bottle or jar? | Motor/Phys. | 0.02 | - | - | - |  | 1.042 | 3.267 | - | - | - | - |
| 157 | Can your child count objects up to five (e.g., fingers, people)? | Cog./Lang. | 0.01 | - | - | - |  | 1.935 | 3.316 | - | - | - | - |
| 158 | Is this child able to do the following: Ask questions that start with, “who,” “what,” “where,” “when”? | Cog./Lang. | 0.016 | - | - | - |  | 1.407 | 3.376 | - | - | - | - |
| 159 | Can your child draw a straight line? | Motor/Phys. | 0 | - | - | - |  | 1.572 | 3.388 | - | - | - | - |
| 160 | Can this child stand on one foot for at least 5 seconds? | Motor/Phys. | 0.029 | - | - | - |  | 1.033 | 3.399 | - | - | - | - |
| 161 | Can your child correctly ask questions using any of the words "what," "which," "where," or "who"? | Cog./Lang. | 0.013 | - | - | - |  | 2.525 | 3.437 | - | - | - | - |
| 162 | Can your child correctly use any of the words "I," "you," "she," or "he" (e.g., "I go to store," or "He eats rice")? | Cog./Lang. | 0 | - | - | - |  | 2.77 | 3.48 | - | - | - | - |
| 163 | Can your child explain in words what common objects like a cup or chair are used for? | Cog./Lang. | 0.016 | - | - | - |  | 2.801 | 3.489 | - | - | - | - |
| 164 | Does your child regularly use describing words such as "fast," "short," "hot," "fat," or "beautiful" correctly? | Cog./Lang. | 0.013 | - | - | - |  | 1.978 | 3.501 | - | - | - | - |
| 165 | If you ask your child to give you three objects (e.g., stones, beans), does the child give you the correct amount? | Cog./Lang. | 0.024 | - | - | - |  | 1.623 | 3.508 | - | - | - | - |
| 166 | Can this child recognize and name emotions in themselves? | Soc. Emot. | 0.027 | - | - | - |  | 1.167 | 3.549 | - | - | - | - |
| 167 | Can this child identify: a square? | Cog./Lang. | 0.021 | - | - | - |  | 1.914 | 3.561 | - | - | - | - |
| 168 | If you point to an object, can your child correctly use the words "on," "in," or "under" to describe where it is (e.g., "The cup is on the table" instead of "The cup is in the table.")? | Cog./Lang. | 0.007 | - | - | - |  | 2.177 | 3.607 | - | - | - | - |
| 169 | Can this child sort objects by: shape? | Cog./Lang. | 0.032 | - | - | - |  | 1.783 | 3.608 | - | - | - | - |
| 170 | Can this child sort objects by: size? | Cog./Lang. | 0.028 | - | - | - |  | 1.894 | 3.611 | - | - | - | - |
| 171 | Can this child show you with their fingers how old they are? | Cog./Lang. | 0.029 | - | - | - |  | 1.618 | 3.614 | - | - | - | - |
| 172 | Can the child indicate when he/she needs to go to the toilet? | Soc. Emot. | 0.007 | - | - | - |  | 1.465 | 3.628 | - | - | - | - |
| 173 | Can this child identify: a triangle? | Cog./Lang. | 0.025 | - | - | - |  | 1.704 | 3.628 | - | - | - | - |
| 174 | Can this child draw a: circle? | Cog./Lang. | 0.021 | - | - | - |  | 1.517 | 3.635 | - | - | - | - |
| 175 | Is this child able to do the following: Ask questions that start with “why” and “how”? | Cog./Lang. | 0.015 | - | - | - |  | 1.68 | 3.711 | - | - | - | - |
| 176 | Can your child say what others like or dislike (e.g., "Mama doesn't like fruit," Papa likes football)? | Soc. Emot. | 0.017 | - | - | - |  | 3.067 | 3.792 | - | - | - | - |
| 177 | Does your child ask "why" questions (e.g., "Why are you tall?")? | Cog./Lang. | 0.016 | - | - | - |  | 2.481 | 3.82 | - | - | - | - |
| 178 | Can your child recognize at least 5 letters of the alphabet? | Cog./Lang. | 0.047 | - | - | - |  | 1.179 | 3.85 | - | - | - | - |
| 179 | Can your child count 10 objects, for example, 10 fingers or blocks, without mistakes? | Cog./Lang. | 0.042 | - | - | - |  | 1.809 | 4.118 | - | - | - | - |
| 180 | Can your child tell a story? | Cog./Lang. | 0.013 | - | - | - |  | 2.196 | 4.119 | - | - | - | - |
| 181 | If you draw a circle can your child do it, just as you did? | Motor/Phys. | 0.029 | - | - | - |  | 1.323 | 4.181 | - | - | - | - |
| 182 | Does your child understand the term 'longest'? For example, if you ask them to choose 'which is the longest of 3 objects?' e.g. 3 spoons or sticks, would he/she be able to choose the longest? | Cog./Lang. | 0.025 | - | - | - |  | 1.883 | 4.25 | - | - | - | - |
| 183 | Can this child read one-digit numbers like 4 or 7? | Cog./Lang. | 0.055 | - | - | - |  | 1.106 | 4.268 | - | - | - | - |
| 184 | Can your child go to the toilet by him/her-self? | Soc. Emot. | 0.01 | - | - | - |  | 1.504 | 4.349 | - | - | - | - |
| 185 | Can this child sort objects by: length? | Cog./Lang. | 0.049 | - | - | - |  | 1.653 | 4.375 | - | - | - | - |
| 186 | Can your child talk about things that will happen in the future using correct language (e.g., "Tomorrow he will attend school" or "Next week we will go to the market")? | Cog./Lang. | 0.01 | - | - | - |  | 2.24 | 4.425 | - | - | - | - |
| 187 | If you had four objects, could this child divide them in half so you have two and they have two? | Cog./Lang. | 0.05 | - | - | X |  | 1.027 | 4.523 | - | - | - | - |
| 188 | Can your child talk about things that have happened in the past using correct language (e.g., "Yesterday I played with my friend" or "Last week she went to the market")? | Cog./Lang. | 0.025 | - | - | - |  | 1.956 | 4.538 | - | - | - | - |
| 189 | Does the child sometimes save things like candy or new toys for the future? | Soc. Emot. | 0.026 | - | - | - |  | 0.739 | 4.561 | - | - | - | - |
| 190 | Can this child draw a face with eyes and mouth? | Motor/Phys. | 0.054 | - | - | - |  | 1.787 | 4.733 | - | - | - | - |
| 191 | Can your child dress him/herself completely (except for shoelaces, buttons and zippers)? | Motor/Phys. | 0.017 | - | - | - |  | 1.227 | 4.898 | - | - | - | - |
| 192 | Can this child draw a: square? | Cog./Lang. | 0.069 | - | - | - |  | 1.578 | 4.994 | - | - | - | - |
| 193 | Can this child draw a: triangle? | Cog./Lang. | 0.079 | - | - | - |  | 1.391 | 5.112 | - | - | - | - |
| 194 | Can this child draw a person with arms and legs? | Motor/Phys. | 0.076 | - | - | - |  | 1.612 | 5.125 | - | - | - | - |
| 195 | Is your child able to go poo or pee without having accidents (wetting or soiling themselves)? | Soc. Emot. | 0.023 | - | - | - |  | 0.922 | 5.548 | - | - | - | - |
| 196 | Can your child fasten and unfasten buttons without help? | Motor/Phys. | 0.031 | - | X | X |  | 0.814 | 5.836 | - | - | - | - |
| 197 | How often can this child correctly subtract two numbers, like 5 take away 2? | Cog./Lang. | 0.056 | X | - | - |  | 0.81 | 5.901 | 7.217 | 8.099 | 9.989 | - |
|  | | | | | | | | | | | | | |

# Kidsights Measurement Tool

## S3 Table. Kidsights Measurement Tool.

| **Item** | **Response Options** |
| --- | --- |
| Does your child cry when he/she is hungry, wet, tired, or wants to be held? | No; Yes |
| Does your child look at and focus on objects in front of him/her? | No; Yes |
| Does your child grasp your finger if you touch his/her hand? | No; Yes |
| Does your child smile? | No; Yes |
| Does your child try to move his/her head (or eyes) to follow an object or person? | No; Yes |
| When lying on his/her back, does your child move his/her arms and legs? | No; Yes |
| Does your child look at a person when that person starts talking or making noise? | No; Yes |
| Does your child hold his/her hands in fists all the time? | No; Yes |
| Does your child make sounds other than crying? | No; Yes |
| Does your child smile when you smile or talk with him/her? | No; Yes |
| Does your child bring his/her hand to his/her mouth? | No; Yes |
| When you talk to your child, does he/she smile, make noise, or more arms, legs or trunk in response? | No; Yes |
| When your child is on his/her stomach, can he/she turn his/her head to the side? | No; Yes |
| When you are about to pick up your child, does he/she act happy or excited? | No; Yes |
| Does your child look at your face when you speak to him/her? | No; Yes |
| Does your child stop crying or calm down when you come to the room after being out of sight, or when you pick him or her up? | No; Yes |
| While your child is on his/her back, can he/she bring his/her hands together? | No; Yes |
| When your child is on his/her stomach, can he/she hold his/her head up off the ground? | No; Yes |
| Does your child make noise or gesture to get your attention? | No; Yes |
| Does your child sometimes suck his/her thumb or fingers? | No; Yes |
| Does your child turn his/her head towards your voice or some noise? | No; Yes |
| Does your child make sounds when LOOKING at toys or people (not crying)? | No; Yes |
| Does your child laugh? | No; Yes |
| Can your child hold his/her head steady for at least a few seconds, without it flopping to the side? | No; Yes |
| Does your child move excitedly, kick legs, move arms or trunk, or make coo noises when a known person enters the room or speaks to them? | No; Yes |
| Does your child grasp onto a small object (e.g., your finger, a spoon) when out in his/her hand? | No; Yes |
| Does your child make single sounds like "buh" or "duh" or "muh"? | No; Yes |
| Does your child try to reach for objects that are in front of him/her by extending one or both arms? | No; Yes |
| When he/she is on his/her tummy, can your child hold his/her head straight up, looking around for more than a few seconds? He/she can rest on his/her arms while doing this. | No; Yes |
| Can your child roll from his/her back to stomach or stomach to his/her side? | No; Yes |
| If you play a game with your child, does he/she respond with interest? For example, if you play peek-a-boo, pat-a-cake, wave bye-bye, etc. does your child smile, widen their eyes, kick or move arms or vocalize? | No; Yes |
| Does your child smile or become excited when seeing someone familiar? | No; Yes |
| Does your child recognize you or other family members (e.g., smile when they enter a room or move toward them)? | No; Yes |
| Does your child show interest in new objects that are put in front of him/her by reaching out for them? | No; Yes |
| Can your child reach for AND HOLD an object, at least for a few seconds? | No; Yes |
| When you put your child on the floor, can she lean on her hands while sitting?  If your child already sits up straight without leaning on her hands, mark 'yes' for this item. | No; Yes |
| When held in a sitting position, can the child hold his/her head steady and straight? | No; Yes |
| Can your child roll from his/her back to stomach, or stomach to back, on his/her own? | No; Yes |
| Can your child eat food from your fingers or off a spoon you hold? | No; Yes |
| Can your child pick up a small object (e.g., a small toy or small stone) using just one hand? | No; Yes |
| If an object falls to the ground out of view, does your child look for it? | No; Yes |
| When lying on his/her stomach, can your child hold his/her head and chest off the ground using only his/her hands and arms for support? | No; Yes |
| When lying on his/her back, does the child grab his/her feet? | No; Yes |
| Can your child sit with support, either leaning against something (furniture or person) or by leaning forward on his or her hands? | No; Yes |
| Is your child interested when he/she sees other children playing? Does she or he watch, smile, or look excited? | No; Yes |
| Does the child look for an object of interest when it is removed from sight or hidden from him/her (e.g., put under a cover, behind another object)? | No; Yes |
| Does your child play by tapping an object on the ground or a table? | No; Yes |
| Does your child intentionally move or change his/her position to get objects that are out of reach? | No; Yes |
| Can your child hold him/herself in a sitting position without help or support for longer than a few seconds? | No; Yes |
| Can your child bang objects together, or bang an object on the table or on the ground? | No; Yes |
| Does your child make two similar sounds together like baba, mumu, pepe, didi (single consonant vowel combinations)? | No; Yes |
| Can your child pass a small object from one hand to the other? | No; Yes |
| Can your child pick up a small object (e.g., a piece of food, small toy or small stone) with just his/her thumb and one finger? | No; Yes |
| Can your child maintain a standing position while holding on to a person or object (e.g., wall or furniture)? | No; Yes |
| While holding onto furniture, does your child bend down and pick up a small object from the floor and then return to a standing position? | No; Yes |
| Can your child pull themselves up from the floor while holding onto something? For example, can they pull themselves up using a chair, a person, or some other object? | No; Yes |
| While holding onto furniture, does your child squat with control (without falling or flopping down)? | No; Yes |
| Can your child pick up and drop a small object (e.g., a small toy or small stone) into a bucket or bowl while sitting? | No; Yes |
| Does your child stop what he/she is doing when you say “Stop!” even if just for a second? | No; Yes |
| Can your child walk several steps while holding on to a person or object (e.g., wall or furniture)? | No; Yes |
| Can your child stand up without holding onto anything, even if just for a few seconds? | No; Yes |
| Can your child maintain a standing position on his/her own, without holding on or receiving support? | No; Yes |
| Can your child make any light marks on paper or in dirt with a crayon or a stick? | No; Yes |
| Can your child pick up small bits of food and feed him/her-self using his/her hand? | No; Yes |
| Can your child climb onto an object (rock, porch, step, chair, bed, low table, etc.)? | No; Yes |
| Can your child take several steps (3-5) forward without holding onto any person or object, even if they fall down immediately afterward? | No; Yes |
| Can your child move around by walking, rather than by crawling on his hands and knees? | No; Yes |
| Does your child make a gesture to indicate “No” (e.g., shaking head)? | No; Yes |
| Can your child stand up from sitting by himself and take several steps forward? | No; Yes |
| Can your child bend down or squat to pick up an object from the floor and then stand up again, without help from a person or object? | No; Yes |
| Can your child make a scribble on paper, or in dirt, in a back and forth manner? For example, can he or she move the pen or pencil or stick back and forth? | No; Yes |
| Can your child follow a simple spoken command or direction without you making a gesture? | No; Yes |
| Can your child fetch something when asked? | No; Yes |
| Can your child drink from an open cup without help? | No; Yes |
| Does your child put his/her hands out to have them washed? | No; Yes |
| While standing, can your child purposefully throw the ball and not just drop it? | No; Yes |
| Can your child walk well, with coordination, without falling down often? With one foot in front of the other (rather than shifting weight side to side, stiff- legged)? | No; Yes |
| Can your child stack at least two objects on top of each other, such as bottle tops, blocks, stones, etc.? | No; Yes |
| Can your child kick a ball or other round object forward using his/her foot? | No; Yes |
| While standing, can your child kick a ball by swinging his/her leg forward? | No; Yes |
| Can your child follow directions with more than one step? For example, "Go to the kitchen and bring me a spoon." | No; Yes |
| Even if your child is unable to do singing games, does he/she enjoy them and want to be a part of them? | No; Yes |
| Can your child stack three or more small objects (e.g., blocks, cups, bottle caps) on top of each other? | No; Yes |
| Can your child run well, without falling or bumping into objects? | No; Yes |
| Can your child greet people either by giving his/her hand or saying “hello”? | No; Yes |
| Does your child share with others (e.g., food)? | No; Yes |
| Does your child dry hands by herself/himself after you have washed them? | No; Yes |
| Is your child able to go poo or pee without having accidents (wetting or soiling themselves)? | No; Yes |
| Can your child say five or more separate words (e.g., names like "Mama" or objects like "ball")? | No; Yes |
| Can your child say ten or more words in addition to "Mama" and "Dada"? | No; Yes |
| Can your child speak using short sentences of two words that go together (e.g., 'Mama go' or 'Dada eat')? | No; Yes |
| Can your child wash hands by him/herself? | No; Yes |
| Can your child ask for something (e.g., food, water) by name when he/she wants it? | No; Yes |
| Can your child break off pieces of food and feed them to him/her-self? | No; Yes |
| Does your child show independence (e.g., wants to pick their own clothes, choose their own activities, or tries to go outside alone)? | No; Yes |
| Can your child correctly name at least one family member other than mom and dad (e.g., name of brother, sister, aunt, uncle)? | No; Yes |
| Can your child walk on an uneven surface (e.g., a bumpy or steep road) without falling? | No; Yes |
| When looking at pictures, if you say to your child "what is this?", can they say the name of the object that you point to? | No; Yes |
| Can your child identify at least seven objects?  For example, when you ask 'where is the ball/spoon/cup/cloth/door/plate/bucket etc.' does your child look at or point to (or even name) the objects? | No; Yes |
| Can your child name at least two body parts (e.g., arm, eye, or nose)? | No; Yes |
| If you show your child an object he/she knows well (e.g., a cup or animal), can he/she consistently name it? | No; Yes |
| Does your child usually communicate with words what he/she wants in a way that is understandable to others? | No; Yes |
| Can your child draw a straight line? | No; Yes |
| Can your child remove an item of clothing (e.g., take off his/her shirt)? | No; Yes |
| Can your child say 15 or more separate words (e.g., names like "Mama" or objects like "ball")? | No; Yes |
| Can your child jump with both feet leaving the ground? | No; Yes |
| Can your child sing a short song or repeat parts of a rhyme from memory by him/herself? | No; Yes |
| Can your child tell you or someone familiar his/her own name/nickname when asked to? | No; Yes |
| Can your child put on at least one piece of clothing by himself? | No; Yes |
| Can your child unscrew the lid from a bottle or jar? | No; Yes |
| Does your child know the difference between the words "big" and "small"?  For example, if you ask, "Give me the big spoon" can your child understand which one to give if there are two different sizes? | No; Yes |
| Can your child speak using sentences of three or more words that go together (e.g., "I want water" or "The house is big")? | No; Yes |
| Can your child correctly use any of the words "I," "you," "she," or "he" (e.g., "I go to store," or "He eats rice")? | No; Yes |
| Can your child say what he/she likes or dislikes (e.g., "I like sweets")? | No; Yes |
| Can your child correctly ask questions using any of the words "what," "which," "where," or "who"? | No; Yes |
| Does your child pronounce most of his/her words correctly? | No; Yes |
| If you show your child two objects or people of a different size, can he/she tell you which one is the big one and which is the small one? | No; Yes |
| Can your child count objects up to five (e.g., fingers, people)? | No; Yes |
| Can your child explain in words what common objects like a cup or chair are used for? | No; Yes |
| If you point to an object, can your child correctly use the words "on," "in," or "under" to describe where it is (e.g., "The cup is on the table" instead of "The cup is in the table.")? | No; Yes |
| Does your child regularly use describing words such as "fast," "short," "hot," "fat," or "beautiful" correctly? | No; Yes |
| Can the child name at least one color (e.g., red, blue, yellow)? | No; Yes |
| Does your child know to keep quiet when the situation requires it? (e.g., at ceremonies, when someone is asleep) | No; Yes |
| Does your child ask "why" questions (e.g., "Why are you tall?")? | No; Yes |
| Can your child tell you when he/she is happy, angry, or sad? | No; Yes |
| If you ask your child to give you three objects (e.g., stones, beans), does the child give you the correct amount? | No; Yes |
| Can your child tell a story? | No; Yes |
| Can your child tell you when others are happy, angry, or sad? | No; Yes |
| Can your child dress him/herself completely (except for shoelaces, buttons and zippers)? | No; Yes |
| Does your child help out around the house with simple chores, even if he/she doesn't do them well? | No; Yes |
| Does your child understand the term 'longest'? For example, if you ask them to choose 'which is the longest of 3 objects?' e.g. 3 spoons or sticks, would he/she be able to choose the longest? | No; Yes |
| Can your child fasten and unfasten buttons without help? | No; Yes |
| If you draw a circle can your child do it, just as you did? | No; Yes |
| Can your child go to the toilet by him/her-self? | No; Yes |
| Can your child stand on one foot WITHOUT any support for at least a few seconds? | No; Yes |
| Can your child say what others like or dislike (e.g., "Mama doesn't like fruit," Papa likes football)? | No; Yes |
| Does your child show respect around elders? | No; Yes |
| Can your child talk about things that have happened in the past using correct language (e.g., "Yesterday I played with my friend" or "Last week she went to the market")? | No; Yes |
| Can your child talk about things that will happen in the future using correct language (e.g., "Tomorrow he will attend school" or "Next week we will go to the market")? | No; Yes |
| Can your child count 10 objects, for example, 10 fingers or blocks, without mistakes? | No; Yes |
| Can your child recognize at least 5 letters of the alphabet? | No; Yes |
| Can your child write his/her own name? | No; Yes |
| Can your child do an activity such as coloring without repeatedly asking for help or giving up too quickly? | No; Yes |
| Does your child get along well with other children? | No; Yes |
| Does your child ask about familiar people other than parents when they are not there, for example, “Where is Grandma?” | No; Yes |
| Does your child offer to help someone who seems to need help? | No; Yes |
| Is this child able to do the following: Understand “in,” “on,” and “under”? | No; Yes |
| How often can this child recognize the beginning sound of a word? For example, the word “ball” starts with the “buh” sound? | None of the time; Some of the time; Half of the time; Most of the time; All of the time |
| When you say a word, how often can this child come up with another word that starts with the same sound? | None of the time; Some of the time; Half of the time; Most of the time; All of the time |
| Does this child repeat or sing rhymes? | No; Yes |
| If you say the word “cat,” how often can this child tell you a word that rhymes with “cat”? | None of the time; Some of the time; Half of the time; Most of the time; All of the time |
| How often can this child explain things they have seen or done so that you understand? | None of the time; Some of the time; Half of the time; Most of the time; All of the time |
| Is this child able to do the following: Ask questions that start with, “who,” “what,” “where,” “when”? | No; Yes |
| Is this child able to do the following: Ask questions that start with “why” and “how”? | No; Yes |
| Can this child sort objects by: color? | No; Yes |
| Can this child sort objects by: shape? | No; Yes |
| Can this child sort objects by: size? | No; Yes |
| Can this child sort objects by: length? | No; Yes |
| How high can this child count correctly? | Child cannot count; Child can count to 5; Child can count to 10; Child can count to 15; Child can count to 20; Child can count to 30 |
| If asked to count objects, how high could this child count correctly? | Child cannot count; Child can count to 5; Child can count to 10; Child can count to 15; Child can count to 20; Child can count to 30 |
| If you had four objects, could this child divide them in half so you have two and they have two? | No; Yes |
| Can this child show you with their fingers how old they are? | No; Yes |
| Can this child read one-digit numbers like 4 or 7? | No; Yes |
| How often can this child correctly add two numbers, like 2 plus 3? | None of the time; Some of the time; Half of the time; Most of the time; All of the time |
| How often can this child correctly subtract two numbers, like 5 take away 2? | None of the time; Some of the time; Half of the time; Most of the time; All of the time |
| Can this child identify: a square? | No; Yes |
| Can this child identify: a circle? | No; Yes |
| Can this child identify: a triangle? | No; Yes |
| If shown two balls, could this child point to the larger ball? | No; Yes |
| Can this child consistently write his or her first name, even if some of the letters aren't quite right or are backwards? | No; Yes |
| How often does this child demonstrate an interest in books by choosing a children's book and turning pages? | None of the time; Some of the time; Half of the time; Most of the time; All of the time |
| How many letters of the alphabet can this child recognize? | None of them; Some of them; Most of them; All of them |
| Can this child draw a: circle? | No; Yes |
| Can this child draw a: triangle? | No; Yes |
| Can this child draw a: square? | No; Yes |
| Can this child feed self with a spoon with little spilling? | No; Yes |
| Can this child make a tower of three or more blocks? | No; Yes |
| Can this child open doors by turning a doorknob or handle? | No; Yes |
| Can this child draw a face with eyes and mouth? | No; Yes |
| Can this child draw a person with arms and legs? | No; Yes |
| When using a pencil, can he or she use fingers to hold it? | No; Yes |
| Can this child climb stairs with one foot on each stair? | No; Yes |
| Can this child jump forward at least six inches? | No; Yes |
| Can this child throw a ball overhand? | No; Yes |
| Can this child catch a large ball with both hands? | No; Yes |
| Can this child stand on one foot for at least 5 seconds? | No; Yes |
| Can this child kick a ball? | No; Yes |
| How often does this child show concern when they see others are hurt or unhappy? | None of the time; Some of the time; Half of the time; Most of the time; All of the time |
| How often does this child play well with others? | None of the time; Some of the time; Half of the time; Most of the time; All of the time |
| Can this child recognize and name emotions in themselves? | No; Yes |
| When necessary, how often does this child listen to adults? | None of the time; Some of the time; Half of the time; Most of the time; All of the time |
| How often does child take turns during games or fun activities? | None of the time; Some of the time; Half of the time; Most of the time; All of the time |
| How often does this child keep working at a task after setbacks? | None of the time; Some of the time; Half of the time; Most of the time; All of the time |
| How often does this child keep working at a task when things don’t work out? | None of the time; Some of the time; Half of the time; Most of the time; All of the time |
| Child bounces back easily when things do not go his/her way. | None of the time; Some of the time; Half of the time; Most of the time; All of the time |
